# Climatic oscillation promoted diversification of spinous assassin bugs during Pleistocene glaciation

**DOI:** 10.1111/eva.13543

**Published:** 2023-03-25

**Authors:** Zhenyong Du, Qian Zhao, Xuan Wang, Teiji Sota, Li Tian, Fan Song, Wanzhi Cai, Ping Zhao, Hu Li

**Affiliations:** ^1^ Department of Entomology, MOA Key Lab of Pest Monitoring and Green Management, College of Plant Protection China Agricultural University Beijing China; ^2^ Sanya Institute of China Agricultural University Sanya China; ^3^ Department of Zoology, Graduate School of Science Kyoto University, Sakyo Kyoto Japan; ^4^ Key Laboratory of Environment Change and Resources Use in Beibu Gulf (Ministry of Education) and Guangxi Key Laboratory of Earth Surface Processes and Intelligent Simulation Nanning Normal University Nanning China

**Keywords:** allopatric speciation, climatic oscillation, diversification, East Asia, glacial refugia, phylogeographic structure, Pleistocene glaciation

## Abstract

Insect speciation is among the most fascinating topics in evolutionary biology; however, its underlying mechanisms remain unclear. Allopatric speciation represents one of the major types of speciation and is believed to have frequently occurred during glaciation periods, when climatic oscillation may have caused suitable habitats to be fragmented repeatedly, creating geographical isolation among populations. However, supporting evidence for allopatric speciation of insects in East Asia during the Pleistocene glaciation remains lacking. We aim to investigate the effect of climatic oscillation during the Pleistocene glaciation on the diversification pattern and evolutionary history of hemipteran insects and to test the hypothesis of Pleistocene species stability using spinous assassin bugs *Sclomina* (Hemiptera: Reduviidae), a small genus widely distributed in southern China but was later found to have cryptic species diversity. Here, using the whole mitochondrial genome (mitogenome) and nuclear ribosomal RNA genes, we investigated both interspecific and intraspecific diversification patterns of spinous assassin bugs. Approximate Bayesian computation, ecological niche modeling, and demographic history analyses were also applied to understand the diversification process and driven factors. Our data suggest that the five species of *Sclomina* are highly diverged, despite three of them currently being cryptic. Speciation occurred during the Pleistocene when suitable distribution areas were possibly fragmented. Six phylogeographic groups in the type species *S. erinacea* were identified, among which two groups underwent expansion during the early Last Glacial Period and after Last Glacier Maximum. Our analyses suggest that this genus may have experienced climate‐driven habitat fragmentation and postglacial expansion in the Pleistocene, promoting allopatric speciation and intraspecific diversification. Our results reveal underestimated species diversity in a small insect group and illustrate a remarkable example of allopatric speciation of insects in East Asia promoted by Pleistocene climatic oscillations. These findings provide important insights into the speciation processes and aid the conservation of insect species diversity.

## 1 INTRODUCTION

How speciation occurs is a central question in evolutionary biology (Foster et al., 1998). Insects represent the most species‐rich animal group, making them an ideal subject for studying the mechanisms driving speciation. Similar to that of other sexual organisms, speciation of insects is a direct consequence of long‐term genetic differentiation (Abbott et al., 2013; Mallet, 2009). Sympatric speciation is mostly promoted by the divergent selection of ecologically relevant traits followed by reproductive isolation (Berlocher & Feder, 2002; Mallet, 2009; Tishechkin, 2020), whereas geographic isolation promotes allopatric speciation owing to mutation, genetic drift, and local adaptation. Sympatric speciation of insects driven by ecological adaptation has been studied extensively (Berlocher & Feder, 2002; Blankers et al., 2019; Cooley et al., 2001; Du, Hasegawa, et al., 2019; Mallet, 2009; Mavárez et al., 2006; Mendelson & Shaw, 2005; Peccoud et al., 2009; Simon et al., 2022; Zhang et al., 2021). As for our understanding of the allopatric speciation of insects, some studies have focused on a local or small geographic scale, such as montane regions and islands, where limited dispersal (of wingless or flightless insects) and local adaptation possibly promote allopatric speciation (Dool et al., 2022; Schat et al., 2022). Many other studies have also investigated the roles of geological processes and climatic changes, such as Pliocene–Pleistocene mountain building and glacial cycles, promoting the allopatric speciation of insects, on larger temporal and geographic scales (Chinn & Gemmell, 2004; Hawlitschek et al., 2012; Ribera & Vogler, 2004; Su et al., 2014).

Pleistocene climatic shifts led to drastic changes in the environment because at least six glaciations occurred during this period (Cox et al., 1977); this subsequently shaped the distribution patterns of species and promoted deep phylogeographic splits within many species (Avise, 2000; Hewitt, 2001; Ji et al., 2020; Ribera & Vogler, 2004; Taberlet, 1998). Meanwhile, areas with complicated topography and local climates provided refugia during glaciation (Petit et al., 2003; Provan & Bennett, 2008). Insects have specialized habitat needs, which have not changed since the Quaternary, as supported by fossil evidence (Elias, 1991). Although climatic oscillations have largely stimulated the evolutionary process and promoted speciation and extinction in many plants and animals (Cristofari et al., 2018; Fontaine et al., 2010; Hewitt, 2000, 2004; Janssens et al., 2009; Tomasello et al., 2020), the findings of Quaternary palaeoentomology studies indicate that species diversity of insects was remarkably stable throughout the glaciation period, especially in northern temperate taxa, which was considered an interesting paradox in the evolutionary legacy of the Ice Ages (Coope, 1970, 1978, 1994, 1995a, 1995b, 2004; Ribera & Vogler, 2004). One possible explanation is that insects have high mobility, allowing them to migrate during periods of climate change; in this case, allopatric populations may not have been isolated for sufficient periods during the glaciations. Although supporting evidence for Pleistocene speciation in insects has been found in the Mediterranean, North America, and Oceania (Chinn & Gemmell, 2004; Hawlitschek et al., 2012; Kaya et al., 2015; Ribera & Vogler, 2004; Vogler et al., 1998), its generality is still unclear particularly in East Asia.

**FIGURE 1:**
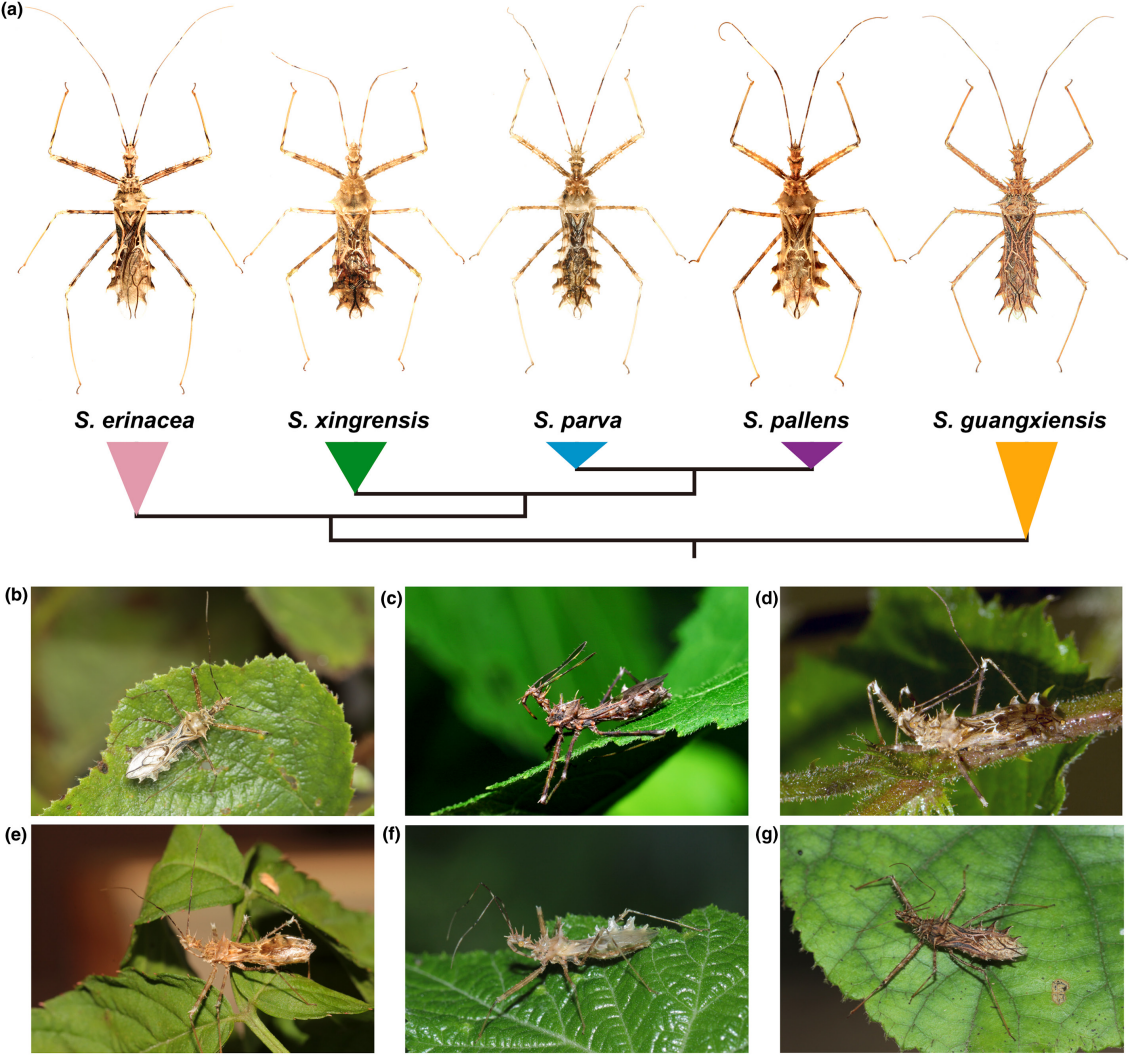
Specimen photos, previously inferred phylogenetic relationships (a), and ecological photos (b–g) of five different *Sclomina* species. (b, c) *S. erinacea*. (d) *S. xingrensis*. (e) *S. parva*. (f) *S. pallens*. (g) *S. guangxiensis*.

*Sclomina*, a small genus in the family Reduviidae, currently contains five species. All of these assassin bugs have a very specific morphology that mimics spiny plants and have been reported to have symbiotic relationships with *Rubus* plants, which makes them great predators and potential biocontrol agents against important agricultural and forestry pests (Zhao et al., 2021). The type species *S. erinacea* Stål was first described in 1861 and its sister species *S. guangxiensis* Ren was described in 2001 based on differences in their morphological traits. Except *S. guangxiensis*, morphological traits were not clearly distinguishable for the identification of more species (Figure 1). Three cryptic species namely *S. parva* P. Zhao and Cai, *S. pallens* P. Zhao and Cai, and *S. xingrensis* P. Zhao and Cai were identified in 2021, mainly based on DNA barcoding sequences (partial mitochondrial *COX1*; Zhao et al., 2021). Except the type species *S. erinacea*, which is widespread in southern China and northern Vietnam, all other four species are narrowly distributed among one or two specific allopatric populations (Zhao et al., 2021). The recent description of cryptic species indicates that species diversity has been long underrated in this genus; thus, elucidating the diversification history of *Sclomina* might provide insights into understanding the diversification process and driving factors of hemipteran insects in East Asia.

Here, we applied large‐scale sampling and next‐generation sequencing to obtain the whole mitochondrial genome (mitogenome), which has been demonstrated to be very promising for resolving recent divergence history (Allio et al., 2017; Du, Hasegawa, et al., 2019; Morin et al., 2010), and used nuclear ribosomal RNA genes as a contrast. We investigated both interspecific and intraspecific diversification patterns of *Sclomina* and applied approximate Bayesian computation (ABC) inference, ecological niche modeling, and demographic analyses to test the hypothesis of Pleistocene species stability and elucidate whether climatic oscillations in the Pleistocene glaciation promoted the allopatric speciation of hemipteran insects in East Asia.

## 2 MATERIALS AND METHODS

### 2.1 Sampling and DNA extraction

In this study, 130 *Sclomina* specimens, collected from 25 sampling sites in China, were used to reconstruct the phylogenetic relationships of this genus (Table S1). Another 124 *S. erinacea* specimens, including 122 collected from the existing 20 sample sites and two collected from two other localities, were added to better resolve the phylogeographic structure of the most widespread type species in *Sclomina* (Table S1). Total genomic DNA was extracted from the thoracic muscles using a TIANamp Genomic DNA Kit (Tiangen Biotech, Beijing, China) following the manufacturer's instructions.

### 2.2 Genome skimming sequencing and assembly

A pooled DNA library with a 350‐bp insert size was built for each of the 254 samples (Du et al., 2021; Gillett et al., 2014; Tang et al., 2014). A total of 6 Gb of 150 bp paired‐end reads were sequenced on the Illumina NovaSeq 6000 platform (Berry Genomics, Beijing, China). Clean reads were trimmed using fastp with reads containing adapter sequences and low‐quality reads (N base number >5, phred quality scores <15 and unqualified percent >40, read length <150) removed (Chen et al., 2018). The previously published mitogenome of *S. erinacea* (GenBank accession MK696614) was used as a reference for generating individual mitogenome sequences. The detailed workflow of the mitogenomic assembly and annotation followed that of Du et al. (2021), where clean reads were mapped onto the reference mitogenome with strict similarity and continuity parameters to generate a consensus sequence for every *Sclomina* individual. The PCR amplification was performed to sequence and validate the repeat regions between *Cytb* and *ND1* for individuals of three species, excluding *S. erinacea* and *S. guangxiensis*, as they could not be assembled well using the above workflow. Three pairs of primers were designed using Geneious Prime 2020 (http://www.geneious.com) based on the reference mitogenome (PCR details are shown in Table S2). The obtained sequences were assembled and aligned with the mitogenomes using Geneious, to complement the previously assembled mitogenomes.

We also assembled the multiple‐copy nuclear ribosomal RNA (rRNA) genes to obtain nuclear phylogenetic trees. De novo assemblies were applied to the libraries of all 130 *Sclomina* individuals using IDBA‐UD (Peng et al., 2012). A “bait” sequence containing partial 5.8S rRNA, internal transcribed spacer (ITS), and 28S rRNA was obtained using the PCR amplification process described above, as a reference. Primer was designed based on Zhang et al. (2015) and listed in Table S2. Local BLAST searches with at least 98% similarity were conducted to obtain the best‐fit scaffolds using the available references. The only one published complete sequence of 18S rRNA, ITS 1, 5.8S rRNA, ITS 2, and 28S rRNA genes of the assassin bug (GenBank accession KM278219) were used to align and annotate the obtained sequences.

### 2.3 Phylogenomic relationship and phylogeographic structure analyses

Phylogenomic and divergence time analyses were performed to reconstruct the phylogenetic relationships of the five *Sclomina* species based on 130 mitogenome and 95 rRNA gene sequences with *Macracanthopsis nodipes* and *Brontostoma colossus* used as outgroups, and to reconstruct phylogeographic structure of *S. erinacea* based on 195 mitogenome sequences with *S. xingrensis* used as outgroup. TranslatorX (Abascal et al., 2010) was used to align 13 protein‐coding genes (PCGs) with the stop codons removed. MAFFT 7.0 was used with the G‐INS‐i strategy (Katoh & Standley, 2013) and the G‐blocks Server (Talavera & Castresana, 2007) to align the rRNA and tRNA genes. Best‐fit schemes and substitution models (Table S3) were selected before the phylogenomic analyses using PartitionFinder 2 (Lanfear et al., 2012) based on the “greedy” algorithm and Akaike information criterion. The 39 codon positions of 13 PCGs, rRNAs, and tRNAs were predefined as separate partitions. The maximum likelihood (ML) and Bayesian inference (BI) methods were used to reconstruct phylogenetic relationships and phylogeographic patterns. The ML analyses were applied using IQ‐TREE 1.6.5 (Trifinopoulos et al., 2016) with 1000 replicates to obtain the bootstrap values for each node. For the BI analyses, two simultaneous Markov chain Monte Carlo (MCMC) runs of 2 million generations were performed using MrBayes 3.2.7 (Ronquist & Huelsenbeck, 2003) to obtain the posterior probability of the nodes. The trees were sampled every 1000 generations with the first 25% discarded as burn‐in. Stationarity was considered reached when the average standard deviation of the split frequencies was below 0.01.

Owing to the lack of fossils for calibration, the molecular clock‐based method was applied in BEAST 2.6.11 (Bouckaert et al., 2014) to estimate the divergence time of the different species and phylogeographic groups. *COX1* was set as a separate partition with the proposed substitution rate of 0.0177 per site per million years in insects (Papadopoulou et al., 2010), whereas the other PCGs, rRNAs, and tRNAs were set as three different partitions with the substitution rates estimated accordingly. In the BEAUti program in BEAST 2, the GTR + I + G model selected by PartitionFinder2 was applied as a site model for each partition. Uncorrelated lognormal relaxed clock and birth–death models were applied as the clock and tree prior models, respectively. Thereafter, two simultaneous MCMC runs of 500 million generations were performed, with trees sampled every 50,000 generations. Tracer 1.7 (Rambaut et al., 2018) was used to verify the stationary distribution of the runs with effective sample sizes of all parameters >200. A consensus tree with “mean height” for each annotated node was generated using the TreeAnnotator program in BEAST 2 after discarding the first 25% of the trees as burn‐in.

MEGA7 (Kumar et al., 2016) was used to calculate the uncorrected genetic distance (*p*‐distance) within and between the five species based on the DNA barcoding sequences (658 bp of *COX1* from the 5′ end). DnaSP 6.0 (Rozas et al., 2017) was used to calculate the number of polymorphic sites (S), number of haplotypes (Nh), haplotype diversity (Hd), and nucleotide diversity (π) and to generate the haplotype data. BAPS 6.0 (Cheng et al., 2013) was used to perform spatial clustering of individual groups. The TCS haplotype networks (Bandelt et al., 1999) were constructed using PopART (Leigh & Bryant, 2015).

### 2.4 Speciation history inference

Approximate Bayesian computation (ABC) method was used to further infer the speciation history of five *Sclomina* species using DIYABC 2.1 (Cornuet et al., 2014). We simulated our mitogenomic datasets with predefined interspecific divergence scenarios and compared these scenarios based on the summary statistics of the observed data. All eight “one sample summary statistics” and five “two sample summary statistics” in this software were applied with the number of simulated data sets recommended by the software. The posterior probability of different scenarios was compared using logistic regression (Fagundes et al., 2007) with linear discriminant analysis (Estoup et al., 2012), and only 1% of the simulated data closest to the observed data were applied in these estimations (Beaumont et al., 2002). Posterior model checking was performed on the selected scenario for each analysis using a local regression based on 1% of the simulated data closest to our data (Beaumont et al., 2002).

To simplify the complicated scenarios among the five species (up to 120 different scenarios), we split the scenario comparison into three different steps, in which the relationship between specific species could be relatively fixed in the following analyses. In step 1, divergence among *S. erinacea*, *S. pallens*, and *S. guangxiensis* was investigated with nine possible scenarios tested. In step 2, *S. parva* was included in the analyses, and eight scenarios were tested. In step 3, *S. xingrensis* was added to the analyses with 14 scenarios tested. The prior values of parameters in all steps are summarized in Table S4.

### 2.5 Demographic history inference

A neutral test and Bayesian skyline plot (BSP) were applied to infer the demographic history of the different species and phylogeographic groups. Arlequin 3.5 (Excoffier & Lischer, 2010) was used to calculate Tajima's *D* (Tajima, 1989) and Fu's *F*s (Fu, 1997) with 1000 simulations to detect bias from the mutation‐drift equilibrium. Confidence intervals of Tajima's *D* and Fu's *F*s were determined by coalescent simulations in DnaSP. BSP analyses were performed using BEAST 2. Partitions and substitution rates were set consistent with the divergence time estimation, except when using the coalescent Bayesian skyline model for tree priors. Two independent MCMC runs of 50 million generations were performed with trees sampled every 1000 generations, discarding 10% of the trees as burn‐in. Tracer software was used to generate BSPs showing historical changes in the effective population size over time.

### 2.6 Ecological niche modeling

Species distribution modeling was used to predict and compare the present and historical distribution areas of *Sclomina* during the Last Glacial Maximum (LGM, 22,000 years ago) and the last interglacial (LIG, 120,000–140,000 years ago) periods. Sample site information from the current study (Table S1) was used as distribution data. Climate data for the present (2.5‐min resolution), LGM (2.5‐min resolution), and LIG (only 30 arc‐seconds resolution available) were downloaded from the WorldClim database (https://www.worldclim.com/version1; Hijmans et al., 2005). To avoid overfitting the models, 19 bioclimatic variables were first filtered based on Pearson's correlation tests (Synes & Osborne, 2011) using IBM SPSS Statistics 19.0 (Chicago, IL, USA). The independent variables that were highly correlated (|*r*| ≥ 0.85) were removed, and the following variables were used for this analysis: BIO1, annual mean temperature; BIO2, mean diurnal range; BIO3, isothermality; BIO5, maximum temperature of the warmest month; BIO8, mean temperature of the wettest quarter; BIO13, precipitation of the wettest month; BIO14, precipitation of the driest month; and BIO15, precipitation seasonality. Using 75% of the present records for training and 25% for model testing, Maxent 3.3.3k (Phillips et al., 2006) was used to predict a suitable distribution with maximum entropy. The parameters used in Maxent are as follows: maximum number of background points = 10,000, regularization multiplier = 1, replicates = 10, replicated run type selected subsample, maximum iterations = 5000, convergence threshold = 10^−5^, and applied threshold rule = 10 percentile training presence. The performance of the model was evaluated based on the area under the curve (AUC) of the receiver operating characteristic (ROC) curve and true skill statistic (TSS). The index of suitability ranged from 0 to 1 and the resulting maps of the predicted suitable distributions were generated using ArcGIS 10.0 (https://www.esri.com/en‐us/arcgis).

### 2.7 Isolation by distance and environment analyses

Isolation by distance (IBD) and isolation by environment (IBE) were tested to investigate potential factors that could drive genetic differentiation among populations of the type species *S. erinacea*. Two different methods, multiple matrix regression with randomization (MMRR) and Mantel test were applied using the R function “MMRR” (Wang, 2013) and ade4 package with 10,000 permutations, respectively. Geographic distances corresponding to straightline (Euclidean) distances were calculated using the R function “dist” and the coordinates of sampling sites. We applied log‐transformed geographic distance to rescale predictor variables. Arlequin was used to calculate pairwise *F*_ST_ values to measure genetic differentiation. The same environmental variables used in the ENM analyses were applied to acquire bioclimatic data for sampling sites, and the first three principal component axes (PC1, PC2, and PC3) explaining 81% of the total variation were used to compute environmental (Euclidian) distances.

## 3 RESULTS

### 3.1 Mitogenomic and nuclear data sets

**FIGURE 2:**
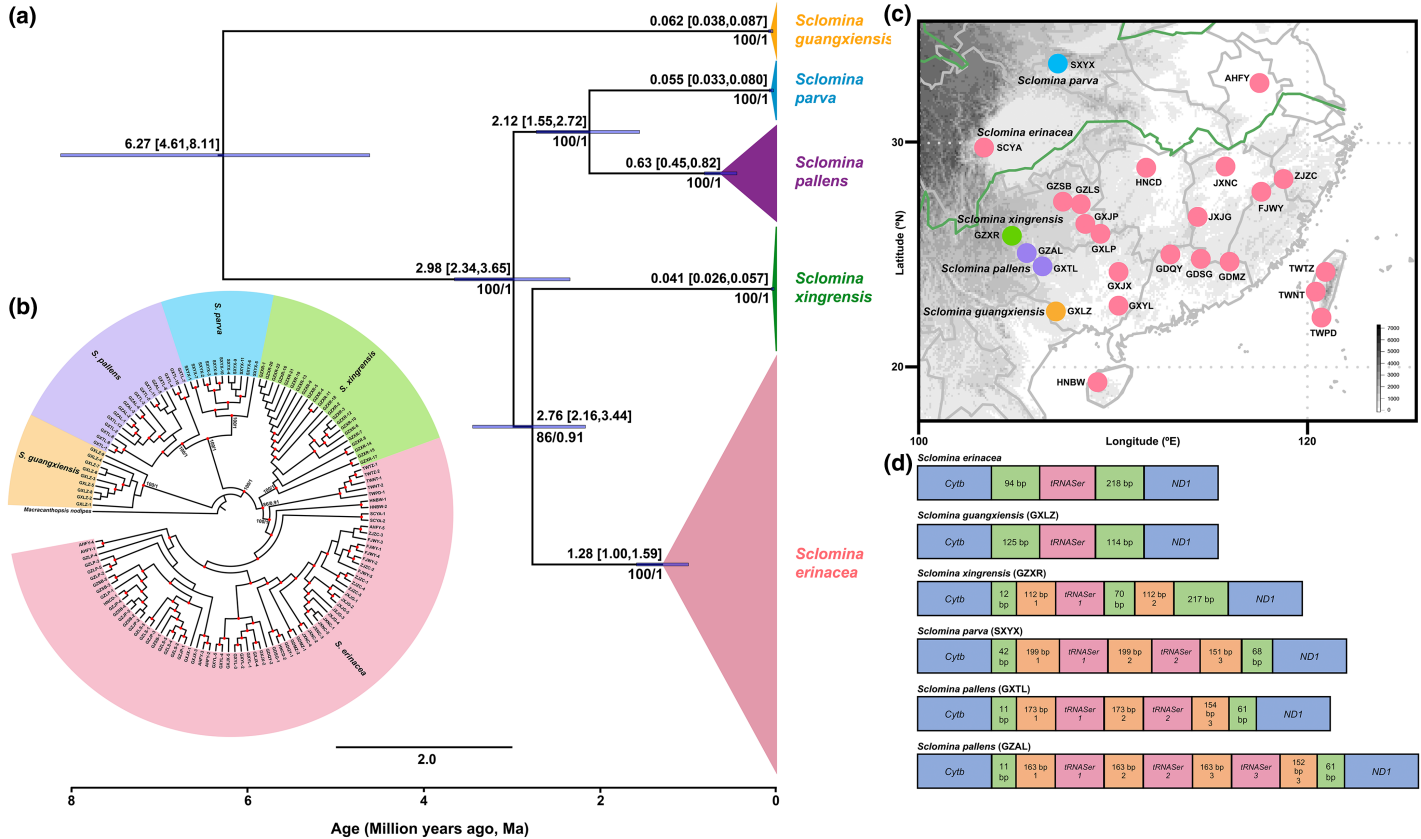
Phylogenetic relationships (a, b) estimated divergence time (a), sampling map (c), and specific mitogenomic features between *Cytb* and *ND1* genes (d) of five *Sclomina* species. The different colors in the phylogenetic trees and sampling map represent different species. (a) The values above the purple bars indicate the mean estimated time with the 95% highest posterior density intervals given in brackets. (b) Topology of this circle tree was reconstructed based on the maximum likelihood (ML) method, with the Bayesian (BI) tree shown in Figure S1. The nodal supports of major branches represent the ML bootstrap percentages and BI posterior probabilities. Red circles represent the other nodes with bootstrap values larger than 70.

In this study, we obtained a total of 254 whole mitogenomes covering all five species of *Sclomina*, among which the reference mitogenomes of *S. guangxiensis*, *S. xingrensis*, *S. pallens*, and *S. parva* were reported for the first time. All functional genes and noncoding regions were sequenced completely, and their lengths ranged from 15,792 to 16,682 bp. All mitogenomes shared the same gene arrangements with the reference mitogenome of *S. erinacea* and the putative ancestral pattern of *Drosophila yakuba* (Clary et al., 1982). The sequence lengths varied among species, which was caused by the length of the noncoding regions among *Cytb*, *tRNA‐Ser*, and *ND1*. These regions were short (120–220 bp) in *S. erinacea* and *S. guangxiensis* and long in *S. xingrensis*, *S. parva*, and *S. pallens* (590–950 bp), with repetitive sequences existing in different patterns (Figure 2d). In *S. xingrensis* (GZXR), two repetitive, 112 bp, noncoding sequences were found with a single *tRNA‐Ser*, whereas in *S. parva* (SXYX) and one *S. pallens* (GXTL) population, three repetitive noncoding sequences were detected with the *tRNA‐Ser* duplicated once. Unexpectedly, in the other *S. pallens* population (GZAL), four repetitive noncoding sequences were detected with the *tRNA‐Ser* duplicated twice (Figure 2d).

For the nuclear rRNA gene data set, 95 sequences with lengths ranging from 1849 to 6985 bp (average length, 4119 bp) were successfully obtained (Table S1). The shortest sequence contained partial 5.8S rRNA, ITS 2, and partial 28S rRNA genes, whereas the longest sequence contained complete 18S rRNA, ITS 1, 5.8S rRNA, ITS 2, and 28S rRNA genes. Additionally, 99.3% of the total alignment sites were identical, which provided very limited information regarding the different species (compared with 74.8% of identical alignment sites in the mitogenomic data set). Therefore, the nuclear rRNA and mitogenomic data sets were analyzed separately in the phylogenetic analyses.

### 3.2 Phylogenomic relationships of *Sclomina*

Based on the concatenated data set of coding genes (14,849 bp in length), the phylogenetic relationships were reconstructed and these strongly supported the monophyly of the five *Sclomina* species (Figure 2, Figure S1). Simultaneously, the maximum likelihood (ML) and Bayesian inference (BI) methods provided a highly consistent resolution of the phylogenetic relationships of the five species (Figure 2, Figure S1), supporting the topology (*S. guangxiensis*, (*S. parva*, *S. pallens*), (*S. xingrensis*, *S. erinacea*)). *Sclomina erinacea* and *S. pallens* showed the highest level of genetic diversity, whereas *S. xingrensis* showed the lowest (Table 1). The genetic distances calculated based on the DNA barcoding sequences were between 0 and 0.93% for within species, and 5.13% and 9.41% for between species (Table S5). The estimated divergence time of *Sclomina* (Figure 2a) suggested that the time of the most recent common ancestor (MRCA) of *Sclomina* was 6.27 million years ago (Ma), with a 95% highest posterior density (HPD) of 4.61–8.11 Ma, at the end of the Miocene. The MRCA time of five different species was more recent and dated between 0.041 and 1.28 Ma, especially for four of the five species, excluding *S. erinacea*, which was dated to 0.041–0.63 Ma, from the middle to late Pleistocene.

**TABLE 1:** Genetic diversity and neutrality test of the five species of *Sclomina* and six phylogeographic groups of *S. erinacea*.

Group	*N*	S	Nh	Hd	π	Tajima's *D* [95% CI]	Fu's *F*s [95% CI]
*S. guangxiensis*	9	80	6	0.833	0.00141	−1.562 [−1.698, 1.593]	3.984 [−2.630, 5.892]
*S. parva*	11	49	6	0.855	0.00094	−0.883 [−1.673, 1.705]	3.820 [−3.749, 4.725]
*S. pallens*	17	519	14	0.978	0.00755	−1.233 [−1.705, 1.543]	3.955 [−3.619, 4.770]
*S. xingrensis*	22	63	11	0.857	0.00082	−1.248 [−1.775, 1.783]	2.720 [−5.454, 6.196]
*S. erinacea*	195	1828	163	0.998	0.01009	−1.728* [−1.561, 1.795]	−21.753* [−19.962, 17.462]
Group 1	11	369	11	1	0.00814	−0.304 [−1.747, 1.592]	0.538 [−0.724, 5.022]
Group 2	2	20	2	1	0.00137	—	2.996 [−3.417, 4.528]
Group 3	5	69	5	1	0.00245	0.567 [−1.215, 1.589]	1.172 [−0.365, 4.920]
Group 4	71	480	61	0.995	0.00272	−2.103* [−1.624, 1.753]	−16.803** [−10.815, 8.577]
Group 5	32	644	27	0.988	0.00696	−1.423 [−1.662, 1.839]	1.272 [−4.896, 5.567]
Group 6	74	506	57	0.991	0.00264	−2.199** [−1.588, 1.793]	−9.843* [−10.961, 10.128]

Abbreviations: CI, confidence interval; Hd, haplotype diversity; *N*, sample size; Nh, number of haplotypes; S, number of polymorphic sites; π, nucleotide diversity.

*p < 0.05, **p < 0.01.

The phylogenetic trees reconstructed based on nuclear rRNA genes showed differences compared with the mitogenomic phylogeny, which supported the topology (*S. guangxiensis*, (((*S. pallen* + *S. xingrensis*), *S. parva*), *S. erinacea*)). The monophyly of three species *S. guangxiensis*, *S. erinacea*, and *S. parva* and the position of *S. guangxiensis* were well supported, whereas *S. pallens* and *S. xingrensis* were clustered together as one clade in the phylogenetic trees (Figures S2 and S3).

### 3.3 Phylogeographic structure of *S. erinacea*

**FIGURE 3:**
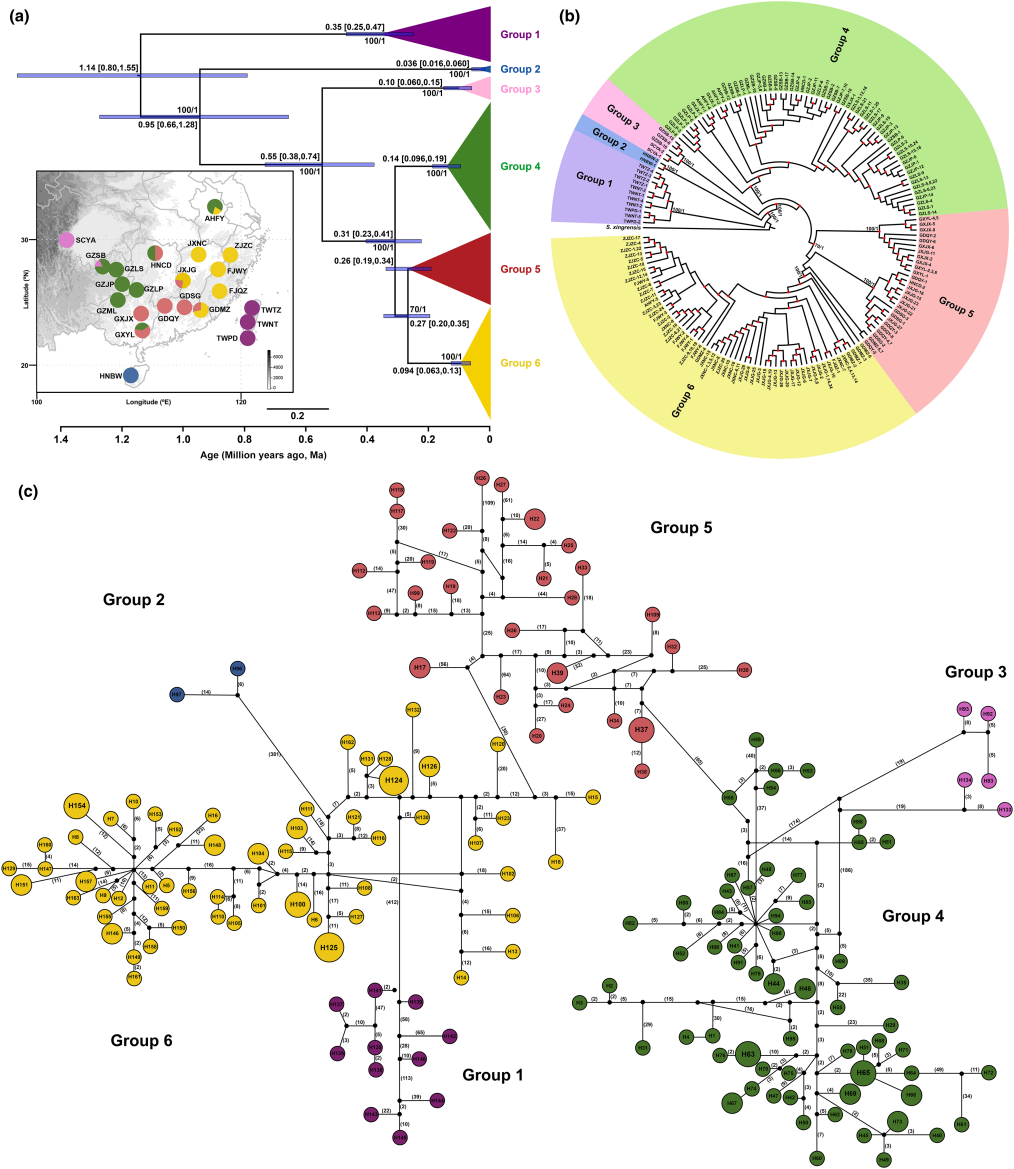
Phylogeographic structure (a, b) estimated divergence time (a) and haplotype network (c) of the type species *Sclomina erinacea*. The different colors in the sampling map and phylogenetic trees represent the six phylogeographic groups resolved by BAPS. (a) The values above the purple bars indicate the mean estimated time with the 95% highest posterior density intervals given in brackets. (b) Topology of this circle tree was reconstructed based on the maximum likelihood (ML) method, with the Bayesian (BI) tree shown in Figure S4. The labels describe the haplotypes shared by individuals, and the nodal supports of major branches represent ML bootstrap values and BI posterior probabilities. Red circles represent the other nodes with bootstrap values larger than 70. (c) The colored circles represent different haplotypes, and the black circles represent missing haplotypes that were not observed. The solid lines between haplotypes represent one mutation step, and the numbers within the brackets represent mutation steps larger than 2. The area of the circle is proportional to the number of haplotypes.

Based on the additional samples and concatenated coding gene data set (14,554 bp in length) of *S. erinacea*, the phylogeographic structure of *S. erinacea* was resolved. The BAPS results divided all individuals into six phylogeographic groups (Groups 1–6, Figure 3a,b), each of which was consistent with one clade in both ML and BI trees, except Group 5, which was close to Group 6 and shown as three separate clades in the trees (Figure 3b, Figure S4). Two of the six phylogeographic groups were located on islands; Group 1 consisted of all populations from Taiwan Island, and Group 2 consisted of populations from Hainan Island (Figure 3). The other four groups were located in mainland China. Group 3 consisted of two populations from around the Sichuan Basin areas, including individuals from populations SCYA and GZSB (Figure 3). Group 4 mainly consisted of individuals from southwestern China (Figure 3). Groups 5 and 6 consisted mostly of individuals from southern and southeastern China, respectively (Figure 3). Among the six phylogeographic groups, nucleotide diversity was highest in Groups 1 and 5 (Table 1). The haplotypes of the mainland groups overlapped in five populations, and the topology (Group 1 (Group 2, (Group 3, (Group 4, (Group 5 + Group 6))))) was highly supported. The haplotype network also supported the close clustering of haplotypes from the same group; the Hd was high, with 163 haplotypes detected in 195 individuals (Figure 3c). Additionally, shared haplotypes were found within the same populations, but not among different populations. Although many haplotypes and mutation steps were missing among the groups, Groups 1 and 2 were found to be more closely connected with Group 6. Group 3 was connected to Group 4, Group 4 to Group 5, and Group 5 to Group 6 in the network relationships (Figure 3c). Divergence time estimation, performed separately for all haplotypes of *S. erinacea* (Figure 3a), showed that the MRCA time of different phylogeographic groups was dated between 0.036 and 0.35 Ma from the middle to late Pleistocene. Notably, Group 5 was considered a single clade to better estimate the divergence time between different phylogeographic groups divided by BAPS, although this was not supported by the phylogenies.

The genetic distances within populations ranged from 0 to 1.56%, whereas those among different populations ranged from 0 to 3.57%. The genetic distances among mainland populations were mostly below 2% (0–1.02%), except for the SCYA population (1.46%–2.55%), while those between mainland‐Taiwan Island (2.17%–3.57%, Table S6) and mainland‐Hainan Island (1.82%–2.66%, Table S6), populations were obviously large.

### 3.4 Speciation history of *Sclomina*

**FIGURE 4:**
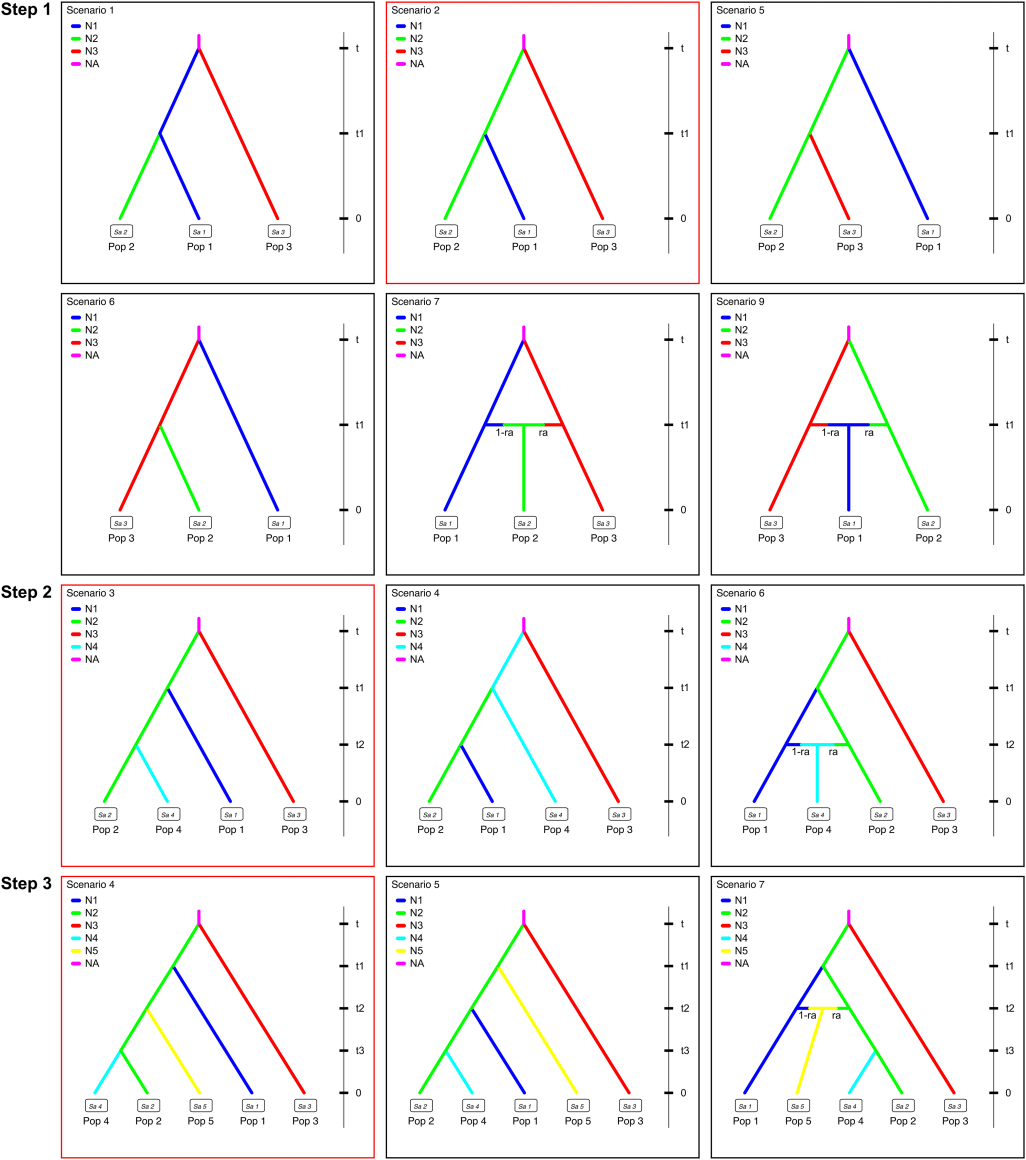
Different scenarios tested in three steps of DIYABC analyses. Pop 1–5 and N1–5 refer to *Sclomina erinacea* (Pop 1), *Sclomina pallens* (Pop 2), *Sclomina guangxiensis* (Pop 3), *Sclomina parva* (Pop 4), *Sclomina xingrensis* (Pop 5) and their effective population sizes, respectively. Only six scenarios in step 1 and three scenarios in steps 2 and 3 with the highest posterior possibility have been shown here. All detailed scenarios of each step are shown in Figures S9–S11. The best scenario in each step was indicated with red squares. Time is not to scale in this figure.

The probability of the selected scenario in ABC analyses is summarized in Table S7, and the model checking results indicated that the simulated data under the selected model fit the observed data (Figures S5–S7, Table S7). In step 1, scenario testing among three species supported a split of *S. erinacea* (Pop 1) from *S. pallens* (Pop 2), and *S. pallens* and *S. guangxiensis* (Pop 3) diverged from an ancestor (Figure 4, Figure S8). In step 2, a later split of *S. parva* (Pop 4) from *S. pallens* than that from *S. erinacea* was supported (Figure 4, Figure S9). In step 3, a split of *S. xingrensis* (Pop 5) from *S. pallens* was supported, which was earlier than that of *S. parva* but later than that of *S. erinacea* (Figure 4, Figure S10). The time for the divergence of *S. guangxiensis* and *S. pallens* was estimated to be 2.08 Ma (5% and 95% quantiles: 0.92, 3.45), and the time for the emergence of *S. erinacea* diverged from *S. pallens* was 1.45 Ma (0.90, 1.92), while those for the emergence of *S. xingrensis* and *S. parva* were 1.01 (0.53, 1.42) and 0.76 Ma (0.32, 1.18), respectively.

### 3.5 Demographic history of *Sclomina*

**FIGURE 5:**
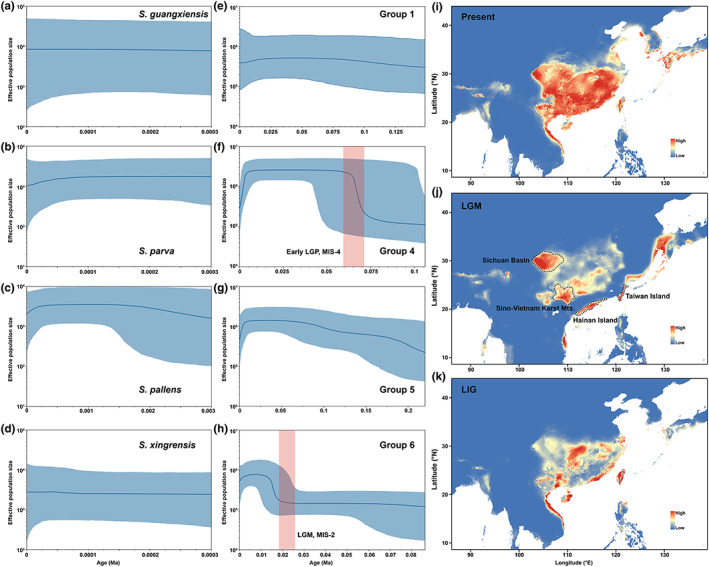
Bayesian skyline plots for different species and phylogeographic groups (a–h) and historical suitable distributions (i–k). (a–h) The mean estimated effective population sizes (lines) are enclosed within the 95% highest posterior density intervals (shaded areas). Orange color areas indicate the time duration for the Last Glacial Maximum (LGM) (Marine Isotope Stage [MIS‐2]) and the Early Last Glacial Period (LGP) (Marine Isotope Stage [MIS‐4]). (i–k) Hindcasting ecological niche models in East Asia during the present (i), LGM (j), and LIG (k) periods. Blue color indicates low suitability and red indicates high suitability. The index of suitability ranged from 0 to 1.

Neutral tests and BSP analyses were applied to infer the demographic history of the different species and groups. Significant negative values were detected in Groups 4, 5, and 6 of *S. erinacea* and in this species when all individuals were treated as a whole, indicating the occurrence of demographic expansion (Table 1). The BSP results (Figure 5a–h) also indicated that expansion occurred in Groups 4, 5, and 6. A significant increase in the effective population size of Group 4 occurred around 0.065–0.07 Ma during the early Last Glacial Period (LGP), that is, during the marine isotope stage (MIS)‐2, after which the effective population size remained steady until a sudden decrease occurred during the last 5000 years (Figure 5f). The effective population size of Group 5 was found to be steadier than that of Group 4, with only a slight increase before 0.05 Ma until a similar decrease occurred recently (Figure 5g). A significant increase in effective population size occurred at 0.012–0.018 Ma in Group 6 (Figure 5h) immediately after the LGM or MIS‐4. Except *S. erinacea*, an effective population size of all other four species was steady without significant changes over time, with only a recent and slight decrease found in both *S. parva* and *S. pallens* (Figure 5a–e).

### 3.6 Ecological niche modeling of *Sclomina*

High AUC (0.992 for present, 0.993 for LGM, and 0.994 for LIG periods) and TSS (0.980 for present, 0.985 for LGM, and 0.976 for LIG periods) values were obtained in the ecological niche modeling (ENM) analyses, supporting the model predictions. Among the environmental variables, BIO13 contributed the most in all models, while BIO5 contributed the least (Table S8). The present suitable distribution areas fit well with the actual distributions of the samples, showing that most areas in southern China are well suited for *Sclomina* (Figure 5i). However, in contrast to the current continuous suitable distribution areas, those predicted for the LGM and LIG periods were contracted and highly fragmented (Figure 5j,k). During the LIG period, the suitable distribution areas for *Sclomina* were mainly located in Central China, the Sino‐Vietnam border, Hainan Island, southeast coastal China, and Taiwan Island (Figure 5k). In the LGM period, the suitable distribution areas were further contracted and located in Sichuan Basin, the Sino‐Vietnam border, narrow areas near Hainan Island, Taiwan Island, and along areas of the East China Sea land bridge up to the Japanese Archipelago (Figure 5j).

### 3.7 Isolation by distance and isolation by environment of *S. erinacea*

The pairwise *F*_ST_ values among 20 populations (*N* > 1) of *S. erinacea* ranged from −0.0223 to 0.9722. The differentiation between the mainland populations and the Taiwan and Hainan Island populations was the highest, ranging from 0.704 to 0.972, while the *F*_ST_ values were also higher between SCYA and other populations, ranging from 0.606 to 0.947 (Table S9). The MMRR results showed that geographic and environmental factors together explained 47.1% variations of the total data. Both MMRR and Mantel test methods revealed that geographical distance and environmental distance significantly correlated with genetic differentiation (MMRR: *β*_D_ = 0.406, *p* = 0.001; *β*_E_ = 0.388, *p* = 0.001; Mantel test: *r*_D_ = 0.405, *p* = 0.001; *r*_E_ = 0.461, *p* = 0.002; Table S10, Figures S11 and S12).

## 4 DISCUSSION

Our data resolved phylogenomic relationships and phylogeographic structure of the spinous assassin bugs *Sclomina* and tested the hypothesis of Pleistocene species stability in East Asia. Climate‐driven habitat fragmentation and postglacial expansion in the Pleistocene may have promoted allopatric speciation and intraspecific diversification. Our results illustrate a remarkable example of the Pleistocene allopatric speciation of hemipteran insects in East Asia and highlight the important roles of climatic oscillations during the Pleistocene glaciation that shaped the diversification patterns of hemipteran insects.

### 4.1 Interspecific and intraspecific divergence

Based on phylogenomic analyses, we further verified the divergence of five species in *Sclomina*, all of which form a well‐supported monophyletic clade. Interestingly, not only the phylogenies but also the features of mitogenomic noncoding regions support the monophyly of the species. In *S. pallens*, the pattern varied between the two separate populations, which indicates their potential isolation and limited gene flow. Notably, the phylogenomic relationships of the five species were different from either our nuclear phylogeny or that reported by a previous study based on DNA barcoding sequences (Zhao et al., 2021). Compared with single mitochondrial gene fragments (e.g., DNA barcoding sequences), those based on whole mitogenomes have a higher resolution and perform better in resolving relationships between closely related species (Du, Hasegawa, et al., 2019; Hirase et al., 2016). The incongruence between mitochondrial and nuclear phylogenies may be caused by the limited resolution of highly conserved rRNA genes. Meanwhile, *S. xingrensis* diverged from *S. pallens* in the ABC analyses, where secondary contact and introgression between *S. xingrensis* and *S. pallens* may also explain the incongruence (Ballard & Whitlock, 2004).

Furthermore, mitogenomic data revealed a clear phylogeographic structure among the widespread *S. erinacea* populations. Six phylogeographic groups were identified in this study. The genetic distances between the two island populations and other populations, especially that between Taiwan Island and other populations, were almost all larger than 2% with some larger than 3%, although these were still less than the interspecific genetic distances of the five species. The high level of divergence observed in the two island groups may make it meaningful to treat them as cryptic allopatric species in the future. The divergence time of these two island groups coincided with the “Ryukyu Coral Sea Stage” (0.2–1.3 Ma) when the sea level rose rapidly and completely submerged the heretofore exposed East China Sea land bridge. The divergence of other mainland phylogeographic groups was consistent with the divisions of Chinese topographical regions (e.g., Sichuan Basin, Guizhou Plateau, Southeast Hills). The topographical boundaries possibly impeded the gene flow of populations and facilitated their differentiation. A similar pattern has also been found in many other hemipteran insects (Du et al., 2021; Du, Ishikawa, et al., 2019; Liu et al., 2018; Zhang et al., 2016). The significant effect of environment on genetic differentiation also indicated the potential adaptation to local climates and provided clues on the climatic sensitivity of these assassin bugs.

### 4.2 Climate‐driven speciation during the Pleistocene

Based on the estimated divergence time, it was surprising to find that although the MRCA time of *S. erinacea* was at around 6.27 Ma during the late Miocene, the MRCA time for the five species occurred much more recently, around 0.041–1.28 Ma during the early to late Pleistocene. The MRCA time of three species, except *S. pallens* and *S. erinacea*, occurred even more recently at 0.041–0.062 Ma during the late Pleistocene, which was later than the MRCA time of four mainland phylogeographic groups (0.094–0.26 Ma) of *S. erinacea*. Besides, the ABC inference inferred the speciation history among five *Sclomina* species, indicating that all three species, except for *S. guangxiensis*, diverged from *S. pallens* successively, and that the earliest divergence between *S. pallens* and *S. guangxiensis* occurred at ~2 Ma, after the start of Pleistocene glaciation. The population size of all species decreased compared with the ancestral population size, which might indicate the potential bottleneck possibly caused by climatic cooling during the Pleistocene glaciation. Although historically suitable distribution area analyses indicated that areas in southern China are highly suitable for *Sclomina* at present, the historical suitable distribution areas were largely contracted into fragmented areas in both the LGM (0.022 Ma) and LIG (0.12–0.14 Ma) periods. It could be inferred that the ancestor of *Sclomina* was widely distributed in southern China before the Pleistocene until drastic climatic cooling occurred with the Pleistocene glaciation, which drastically affected *Sclomina* populations by dividing them into fragmented habitats.

### 4.3 Glacial refugia preserved species diversity

Interestingly, ecological niche modeling analyses resolved the major historical suitable distribution areas during the LGM period were consistent with the ranges of the Sichuan Basin. These three cryptic species (*S. xingrensis*, *S. parva*, and *S. pallens*) were also found located in the surrounding areas of the Sichuan Basin, showing a radial distribution pattern. Moreover, Group 3 of *S. erinacea*, which is distributed in the Sichuan Basin, has a sister relationship with the MRCA of all other mainland phylogeographic groups. This indicated that the mainland population of this species may have originated from these areas. These results indicate that the Sichuan Basin possibly represents one of the potential refugia for *Sclomina* during the Pleistocene glaciation. Previous studies have demonstrated that the Sichuan Basin and the surrounding forests functioned as refugia during glacial periods in China (Song et al., 2018). These areas are distributed in the eastern Himalayas and have long been considered a global biodiversity hotspot harboring many organisms (Wang et al., 2021). It could be inferred that during the LGM period, the mainland populations of *Sclomina*, except *S. guangxiensis*, were possibly distributed around the refugia and survived extinction until the glacial period ended. Furthermore, out‐of‐refugia recolonization after the LGM (0.018–0.026 Ma) can be inferred for *S. erinacea* because a significant expansion was detected in Group 6 immediately after the LGM ended (0.012–0.018 Ma). The phylogenetic trees and haplotype network clearly resolved the connection and direction of mutations from Groups 3 to 6 through Groups 4 and 5. The mitogenomic phylogenetic relationship of *S. parva* and *S. pallens* also provided clues regarding the refugia role of the Sichuan Basin. Although the current distributions of these two species are located far from each other, they were always clustered together as sister groups on the mitochondrial phylogenies (Zhao et al., 2021). It may be inferred that the common ancestor of *S. parva* and *S. pallens* diverged during the recolonization out of refugia. In addition to the Sichuan Basin, the Sino‐Vietnam areas with karst mountains and seasonal forests, Taiwan Island, and Hainan Islands may have functioned as refugia for *Sclomina*, which may account for the deep divergence of *S. guangxiensis*, Group 1 (Taiwan Island), and Group 2 (Hainan Island) in the phylogenetic trees. The populations spread to these islands during land bridge formation and were later isolated when the land bridge became submerged due to sea level rise (Qi et al., 2014; Zhang et al., 2016).

### 4.4 Insight into the Pleistocene species stability and conservation of insects

Quaternary biologists have raised an evolutionary paradox of the Ice Ages for insects, given their high environmental sensitivity to climatic and habitat changes. Insects seem to maintain remarkable stability during the glaciation period, which is supported by fossil evidence (Coope, 1970, 1978, 1994, 1995a, 1995b, 2004). Coope (1994) argued that the high mobility of insects enabled them to respond quickly to sudden climatic changes, which again mixed the genetic pools of populations and eliminated genetic differentiation and further speciation (Coope, 1994, 2004). However, in our study, allopatric speciation was detected along with the possible local extinction of populations of these cryptic allopatric species, which was partially supported by the early divergence but much later MRCA time of different species. These results support the notion that climatic changes and habitat fragmentation led to speciation and extinction events among this insect group. However, the refugia played an important role in defending against extinction and preserving species diversity. The similar local environment in the refugia possibly led to the parallel selection on physiological and ecological traits, which might account for the morphological stasis. This renders it difficult for taxonomists and paleontologists to identify the different species in the absence of genetic data, which would impede the estimation and conservation of species diversity of insects.

## 5 CONCLUSION

Based on the well‐resolved phylogenomic relationships and phylogeographic structure of the spinous assassin bugs *Sclomina*, we verified the divergence of its five species and identified six phylogeographic groups in the type species *S. erinacea*. We found species‐specific mitogenomic features in repetitive noncoding regions and tRNA genes, which also provided evolutionary evidence of speciation. Although mitogenomic coding genes are usually under strong sweeping selection pressure because of their crucial roles in cellular respiration (Du, Hasegawa, et al., 2019), the noncoding regions may have experienced considerably less selection pressure to ensure that the variations in these regions were fixed quickly during evolution. Our study further supported the notion that mitogenomic features could provide important insight into allopatric speciation, which have mostly been ignored in previous studies. These findings contribute to our understanding of the processes underlying the Pleistocene allopatric speciation of insects and emphasize the importance of investigating the diversification patterns of small insect groups whose species diversity might have been greatly underestimated.

## CONFLICT OF INTEREST STATEMENT

The authors declare that they have no conflict of interest.

## Supporting information


Appendix S1

## Data Availability

The sequence data underlying this study have been deposited in GenBank under the accession numbers ON116694–ON116947 (mitogenomes) and ON130390–ON130484 (nuclear rRNA genes). See Table S11 for detailed isolate information. Tree files and sequence alignment matrices are deposited in Dryad: https://doi.org/10.5061/dryad.fxpnvx0x4.
