# Supplementary material for: Climatic oscillation promoted diversification of spinous assassin bugs during Pleistocene glaciation
Source: Evol Appl. 2023 Mar 25;16(4):880–94. doi: 10.1111/eva.13543 (PMC10130555; doi:10.1111/eva.13543)
Supplement: Supplementary file 1 — Appendix S1 [file EVA-16-880-s001.docx]

**Supplementary Information**

**Climatic oscillation promoted diversification of spinous assassin bugs during Pleistocene glaciation**

**Running title:** Pleistocene Speciation of Spinous Assassin Bugs

Zhenyong Du^1,2,†^, Qian Zhao^1,2,†^, Xuan Wang^1,2^, Teiji Sota^3^, Li Tian^1,2^, Fan Song^1,2^, Wanzhi Cai^1,2^, Ping Zhao^4,^*, Hu Li^1,2,^*

*^1^* *Department of Entomology, MOA Key Lab of Pest Monitoring and Green Management, College of Plant Protection, China Agricultural University, Beijing 100193, China*

*^2^ Sanya Institute of China Agricultural University, Sanya 572025, China*

*^3^ Department of Zoology, Graduate School of Science, Kyoto University, Sakyo, Kyoto, Japan*

*^4^ Key Laboratory of Environment Change and Resources Use in Beibu Gulf (Ministry of Education) and Guangxi Key Laboratory of Earth Surface Processes and Intelligent Simulation, Nanning Normal University, Nanning 530001, China*

^†^These authors contributed equally to this work.

***Corresponding authors:**

Hu Li:

No.2 Yuanmingyuan West Road, Beijing 100193, China. Email: tigerleecau@hotmail.com

Ping Zhao

No.175 East Mingxiu Road, Nanning 530001, China. Email: zpyayjl@126.com

**
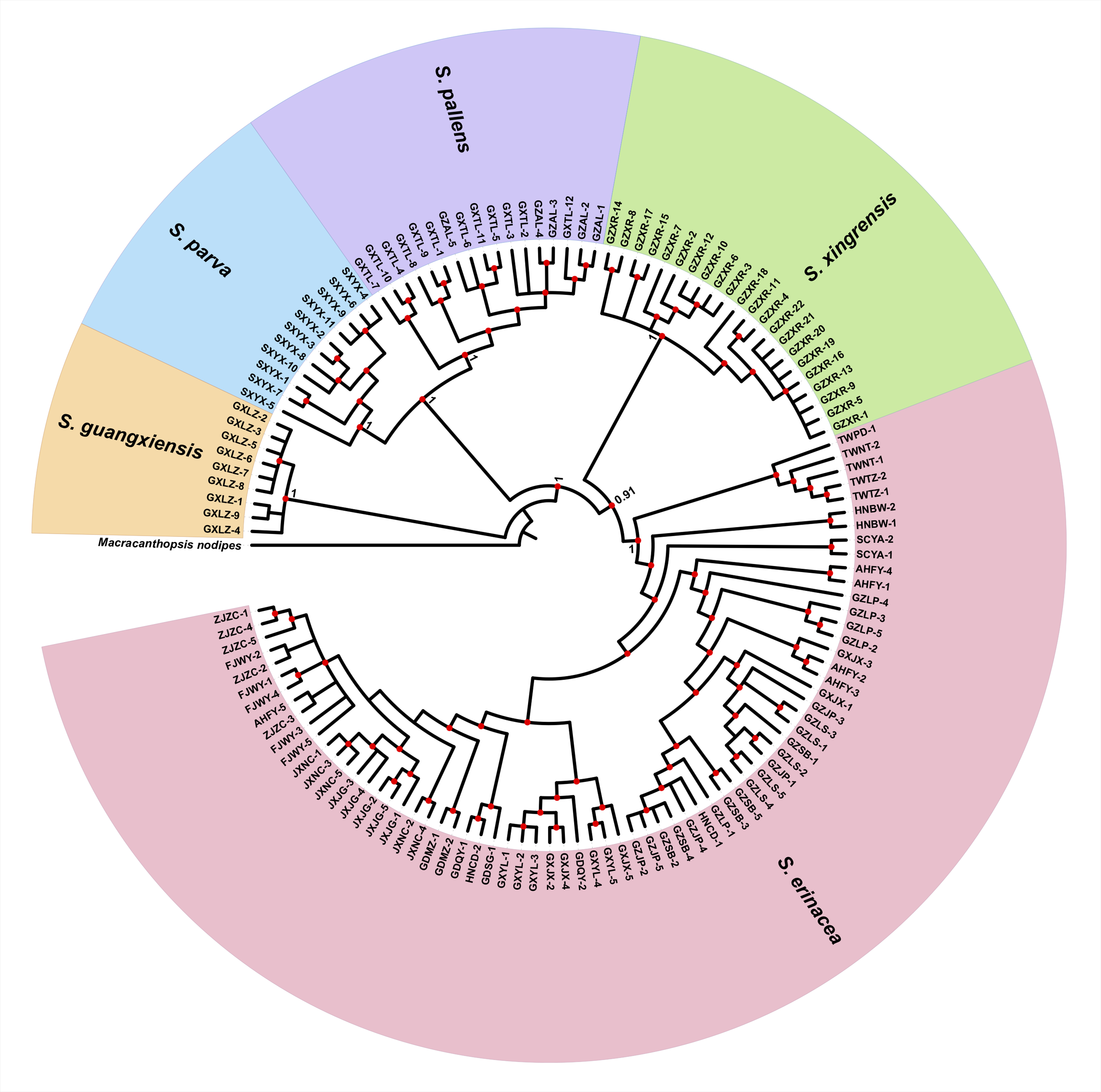
**

**Fig. S1.** The mitogenomic phylogenetic relationships of five *Sclomina* species resolved by MrBayes. The nodal supports of major branches are BI posterior probabilities. Red circles represent the other nodes with posterior probabilities larger than 0.7.

**
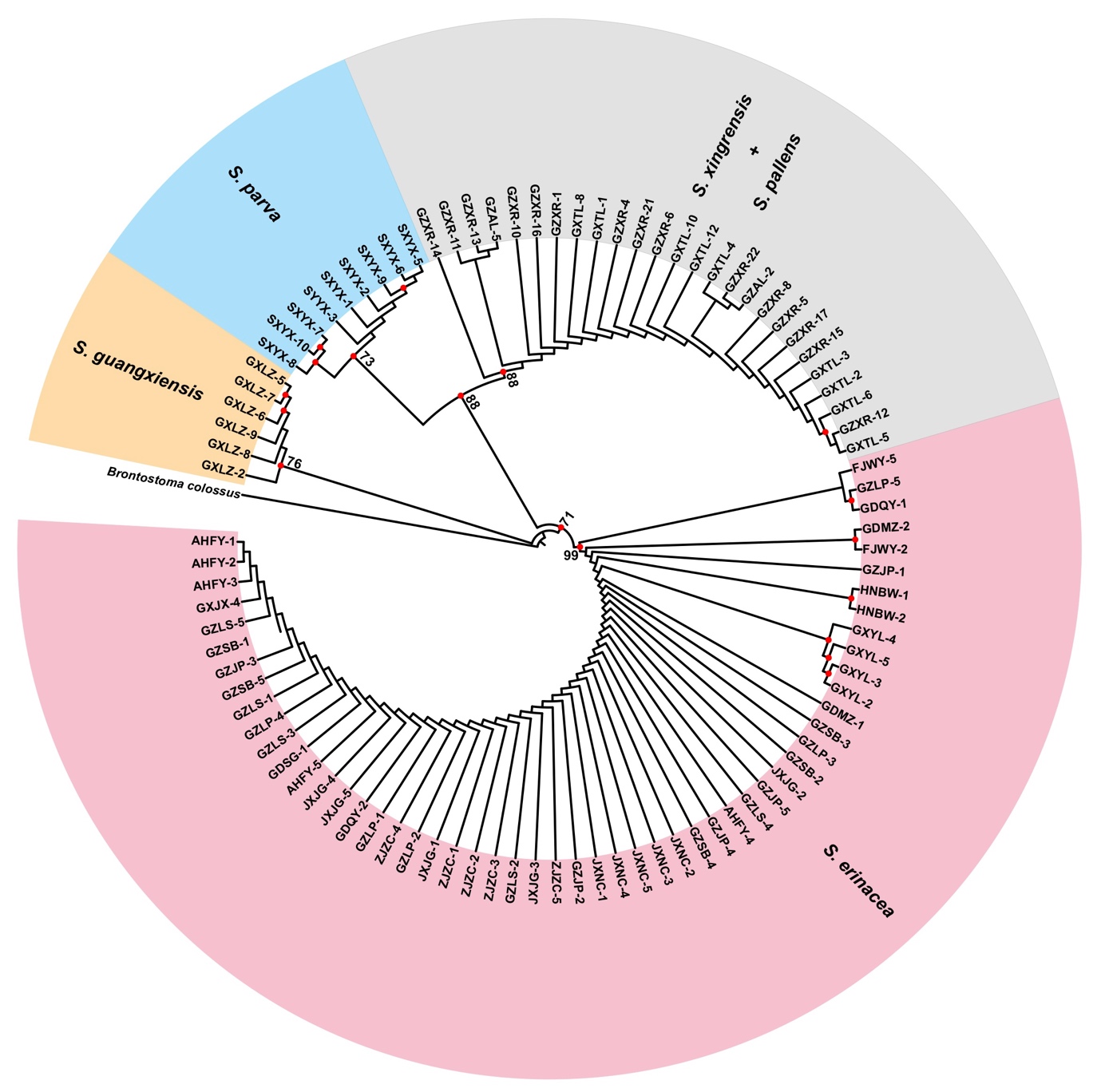
**

**Fig. S2.** The nuclear phylogenetic relationships of five *Sclomina* species resolved by IQ-TREE. The nodal supports of major branches are ML bootstrap values. Red circles represent the other nodes with bootstrap values larger than 70.

**
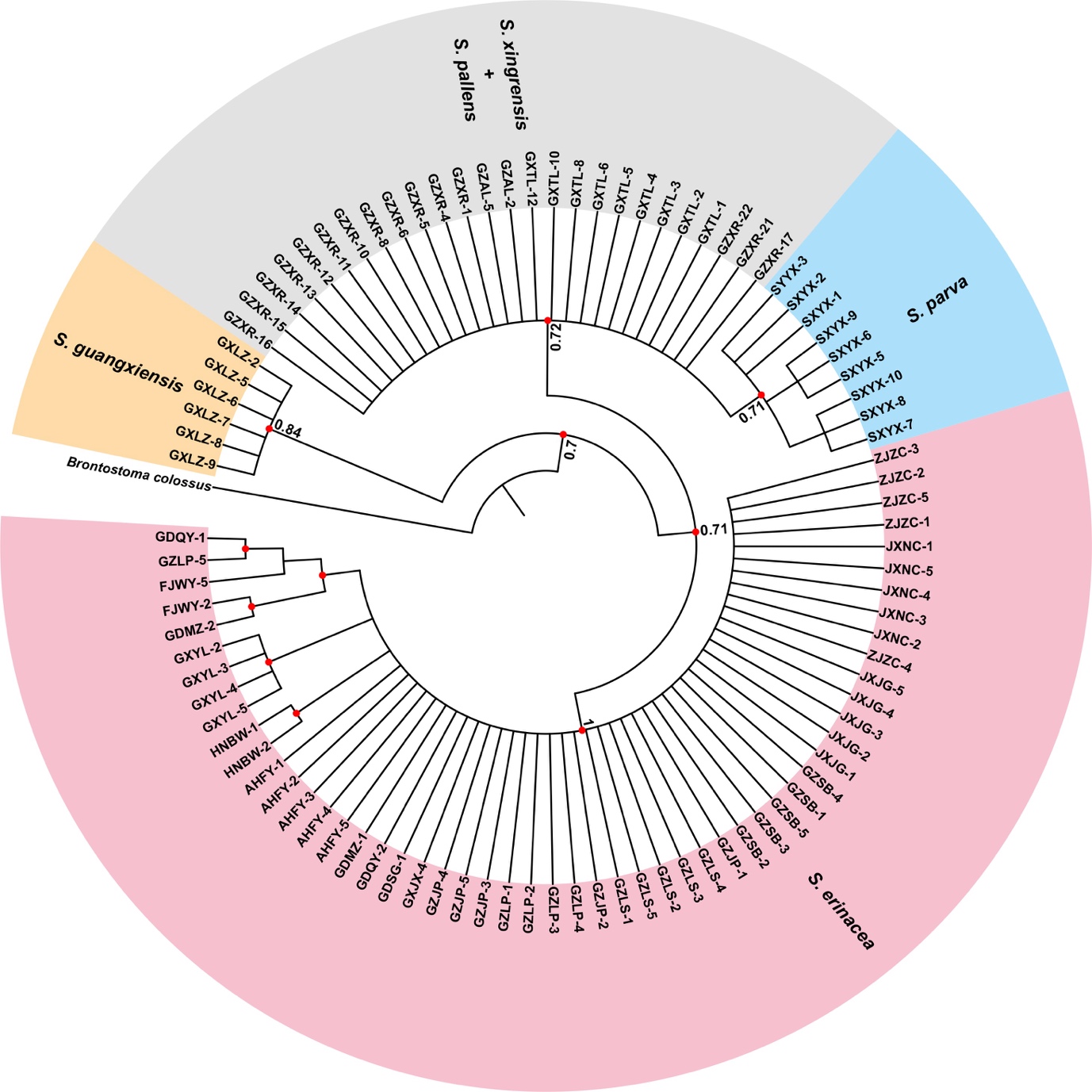
**

**Fig. S3.** The nuclear phylogenetic relationships of five *Sclomina* species resolved by MrBayes. The nodal supports of major branches are BI posterior probabilities. Red circles represent the other nodes with posterior probabilities larger than 0.7.

**
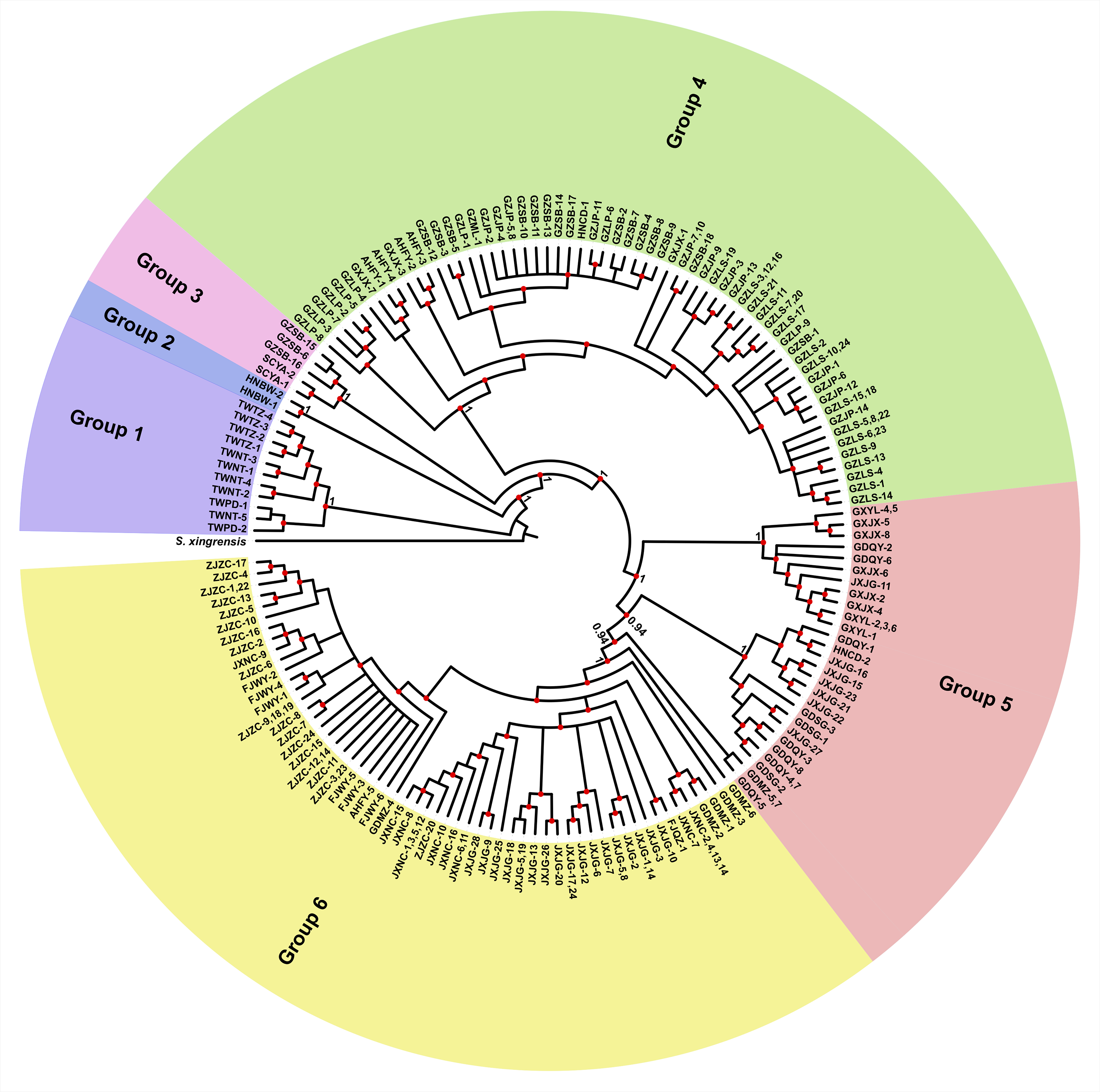
**

**Fig. S4.** The mitogenomic phylogeographic pattern of *S. erinacea* resolved by MrBayes. The labels describe haplotypes shared by individuals, and the nodal supports of major branches are BI posterior probabilities. Red circles represent the other nodes with posterior probabilities larger than 0.7.

**
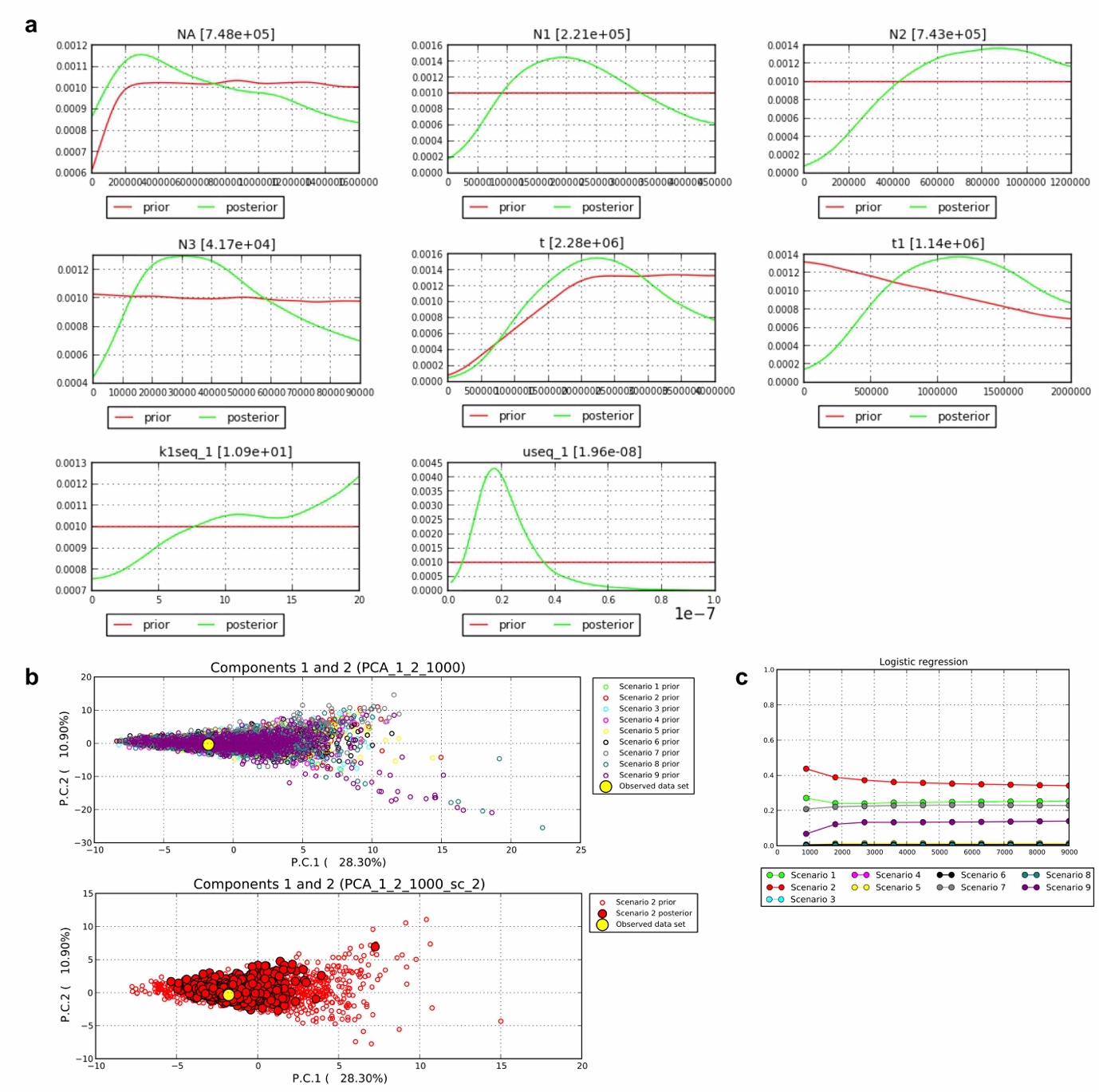
**

**Fig. S5.** The posterior probability of parameters, pre-evaluation of scenario priors and logistic regression plot showing the posterior probability of each scenario in step 1 from the DIYABC analysis.

**
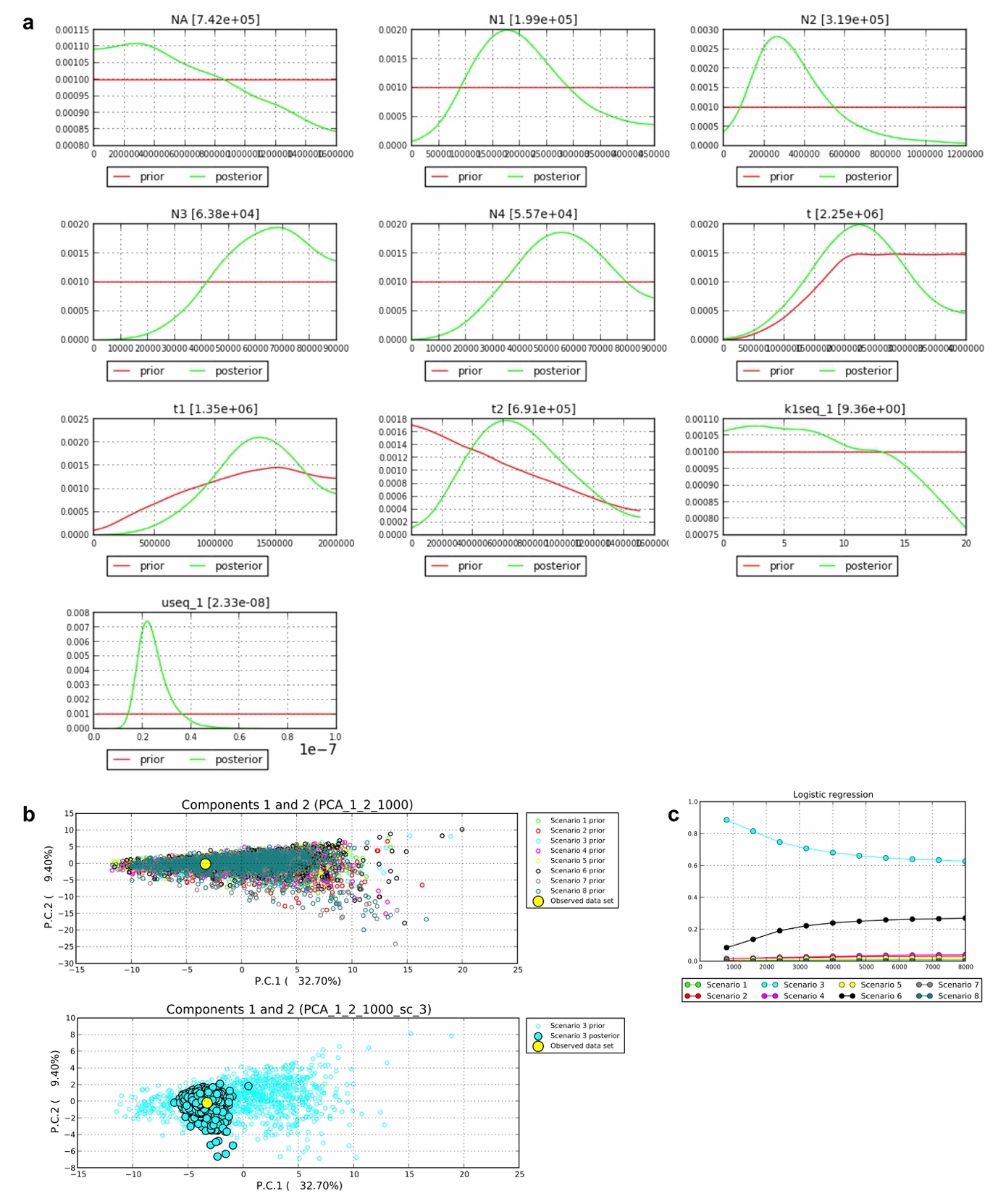
**

**Fig. S6.** The posterior probability of parameters (a), pre-evaluation of scenario priors and model checking for the selected scenario (b), and logistic regression plot (c) showing the posterior probability of each scenario in step 2 from the DIYABC analysis.

**
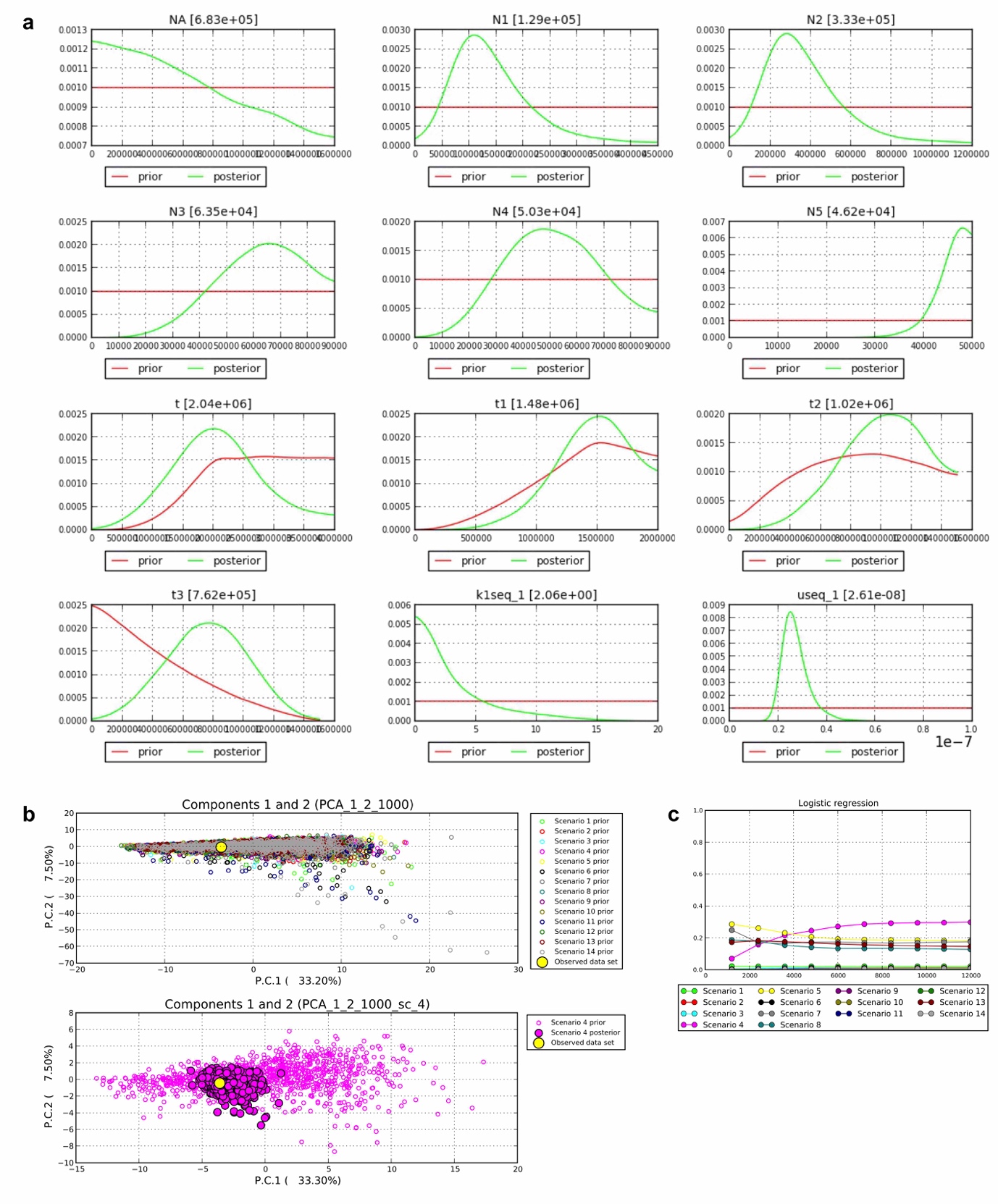
**

**Fig. S7.** The posterior probability of parameters (a), pre-evaluation of scenario priors and model checking for the selected scenario (b), and logistic regression plot (c) showing the posterior probability of each scenario in step 3 from the DIYABC analysis.


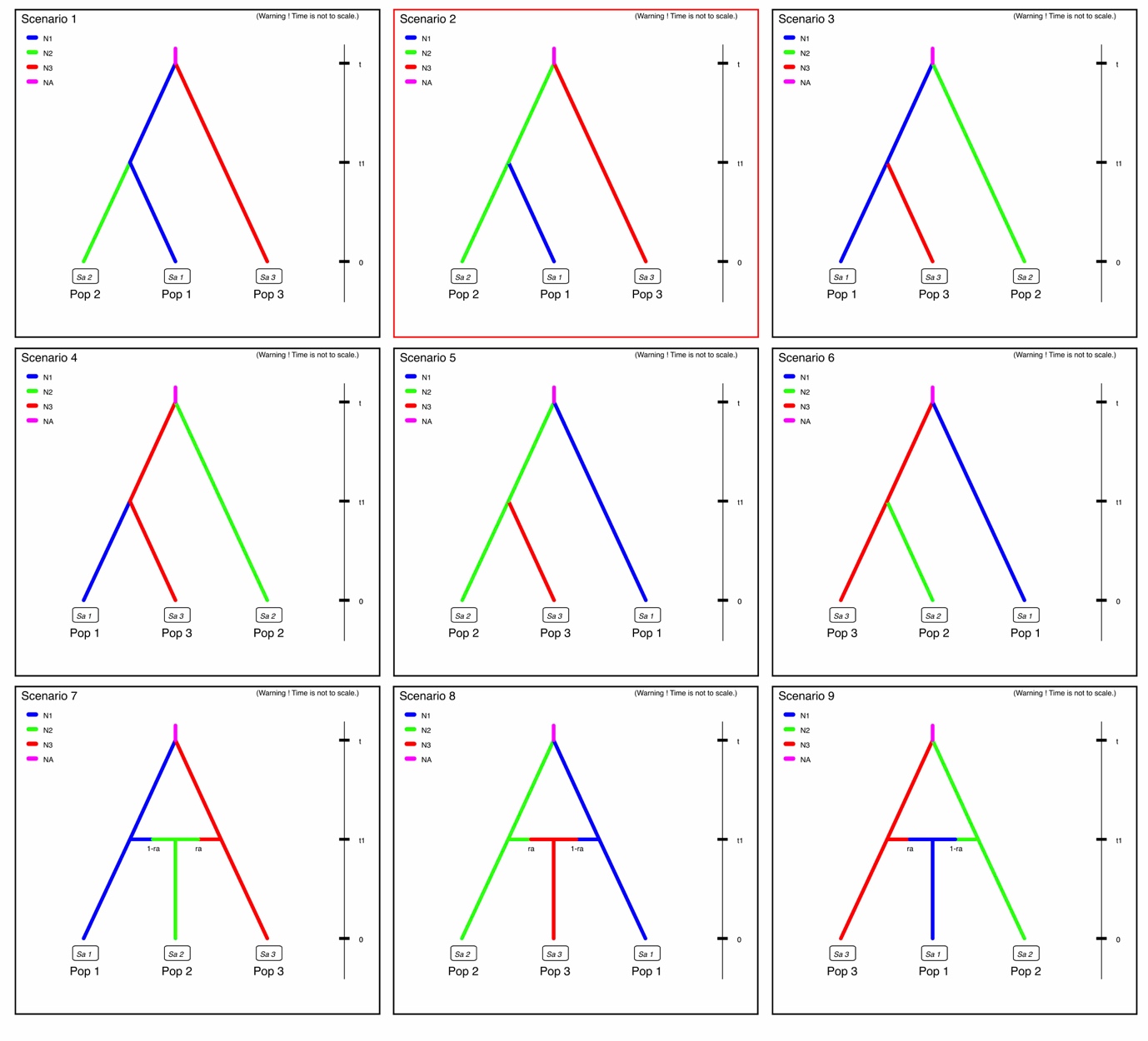


**Fig. S8.** The nine detailed competing scenarios of step 1 in the DIYABC analysis. The best scenario was indicated by red square.

**
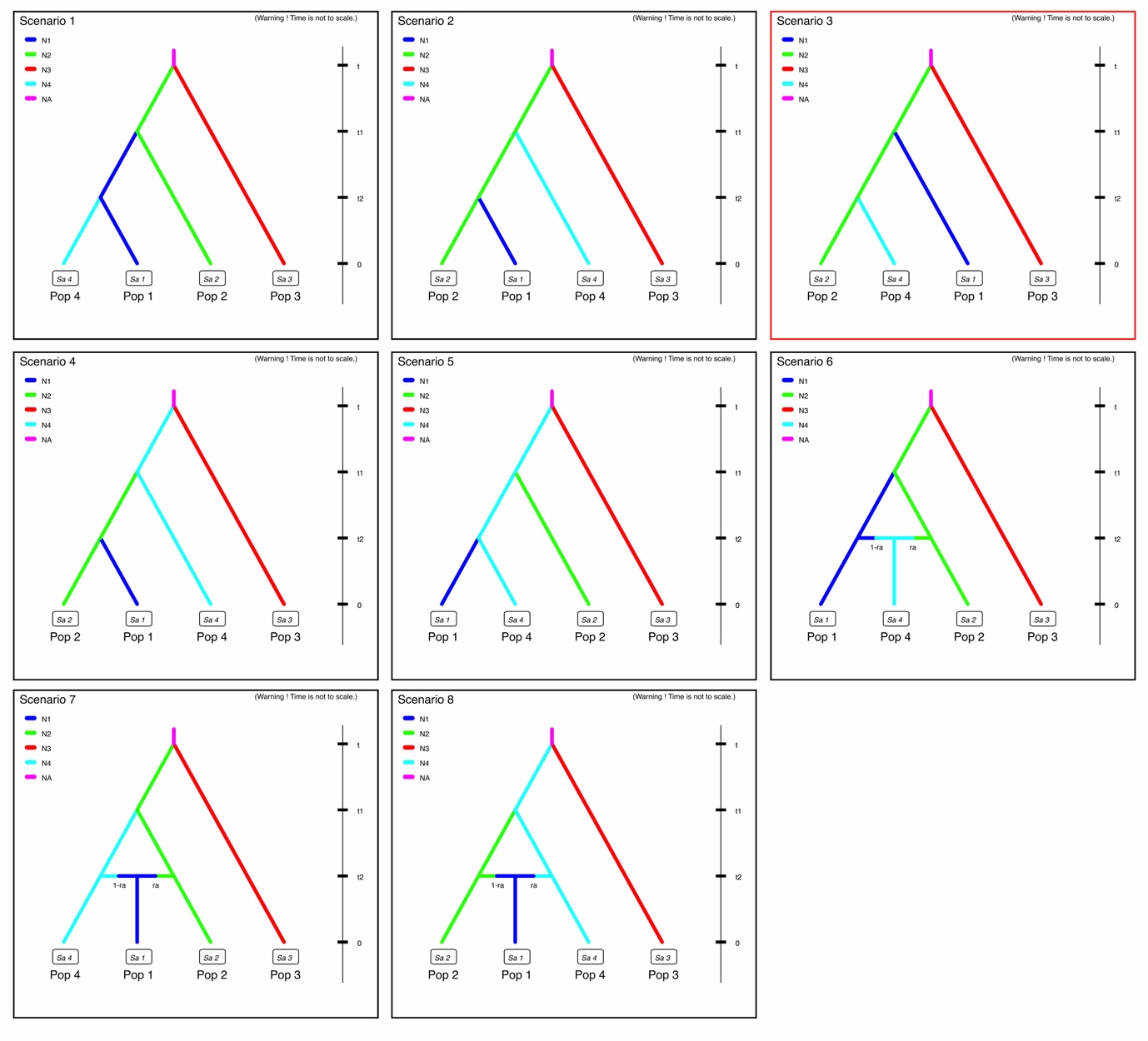
**

**Fig. S9.** The eight detailed competing scenarios of step 2 in the DIYABC analysis. The best scenario was indicated by red square.

**
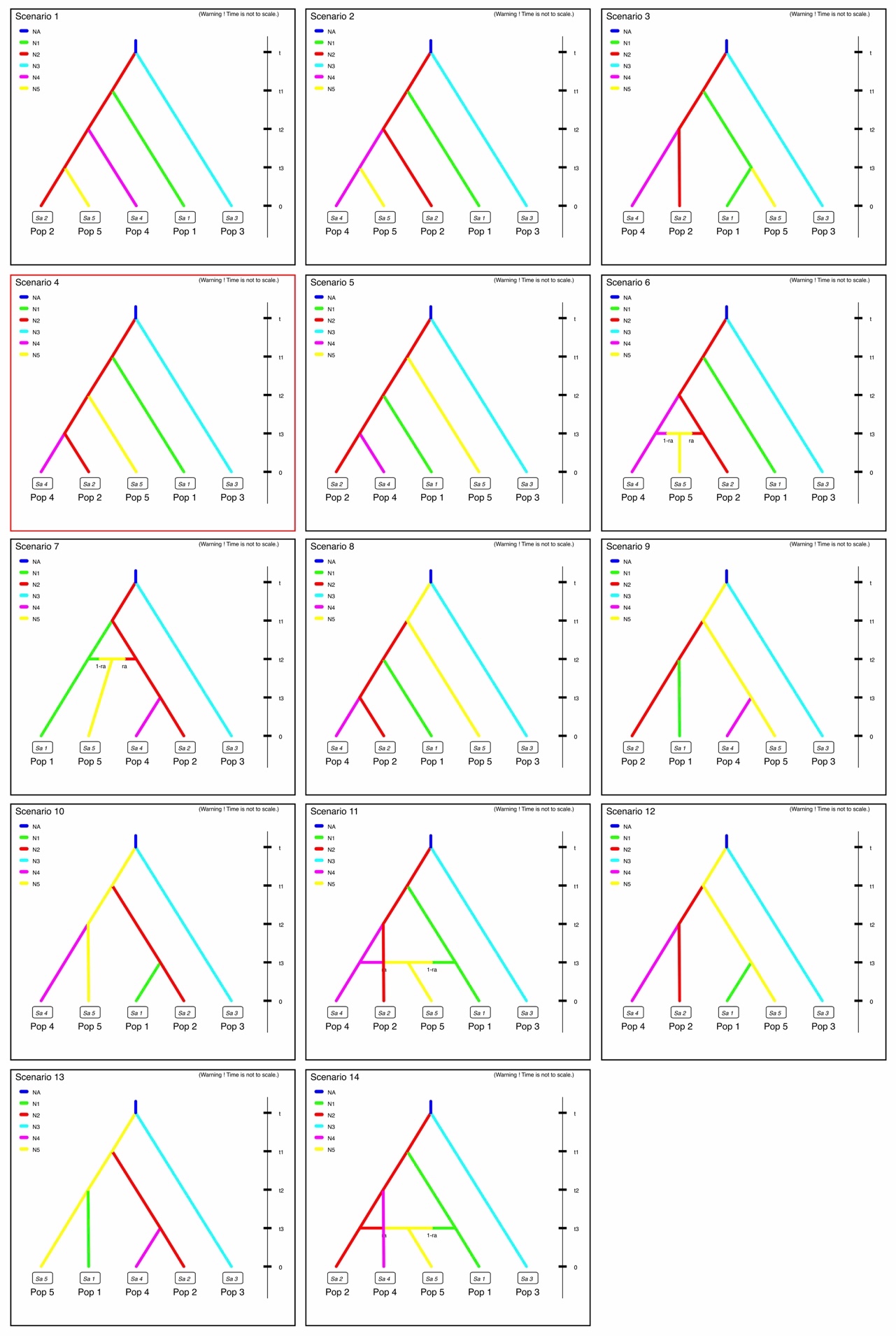
**

**Fig. S10.** The fourteen detailed competing scenarios of step 3 in the DIYABC analysis. The best scenario was indicated by red square.


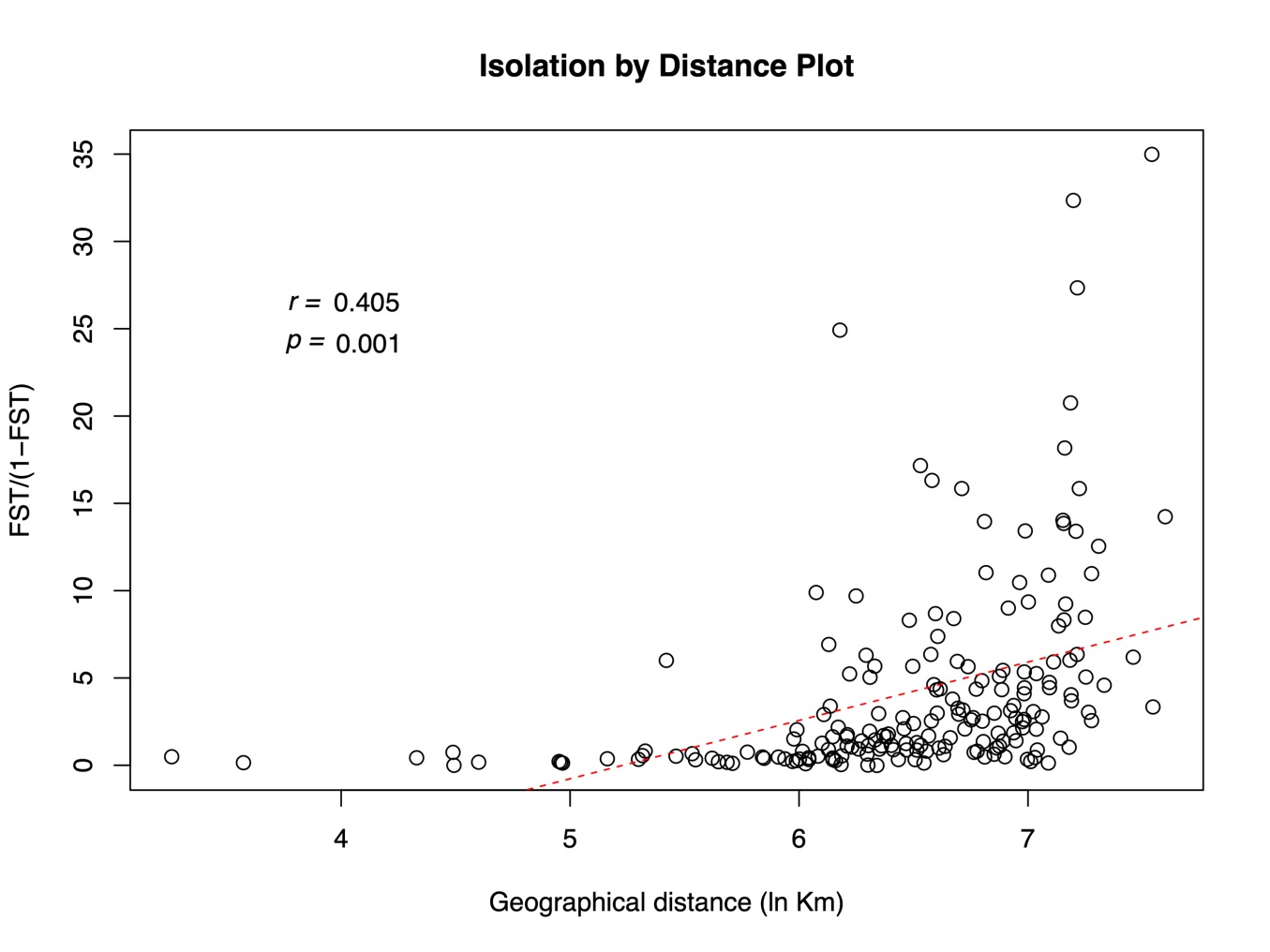


**Fig. S11.** Mantel tests between geographic distance and genetic differentiation in *S. erinacea* populations.

**
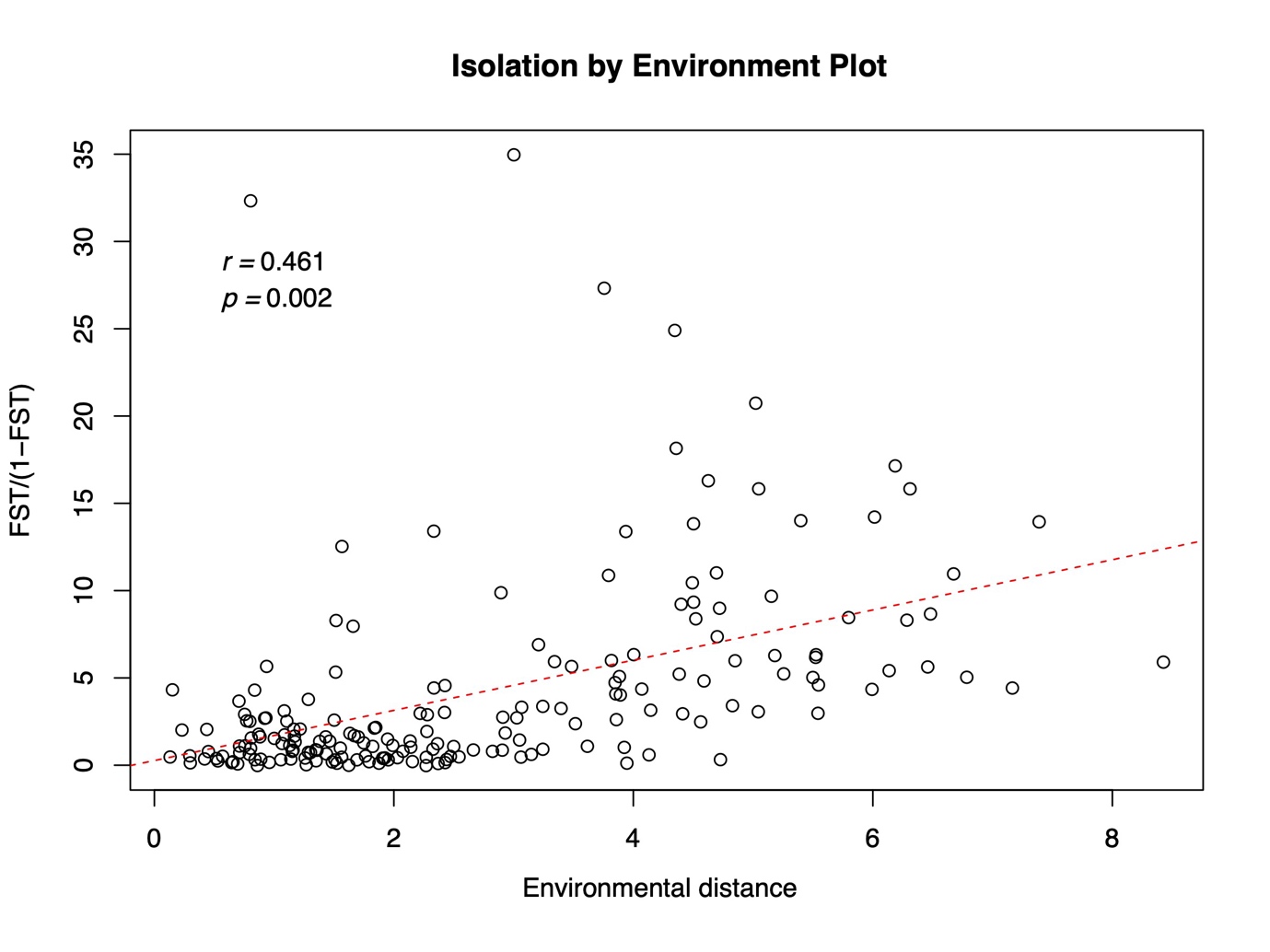
**

**Fig. S12.** Mantel tests between environmental distance and genetic differentiation for *S. erinacea* populations.

**Table S1.** Sample localities of *Sclomina* used in this study.

| Species | Sample sites | Population codes | Collection dates | Longitude (E) | Latitude (N) | Samples | Isolates used in mitogenomic phylogenetic analyses | Isolates obtained in nuclear phylogenetic analyses | Isolates used in population genetic analyses |
| --- | --- | --- | --- | --- | --- | --- | --- | --- | --- |
| *Sclomina erinacea* | Mt. Fengyang, Chuzhou, Anhui | AHFY | 06/05/2008 | 117.57 | 32.66 | 5 | AHFY-1–5 | AHFY-1–5 | AHFY-1–5 |
|  | Mt. Wuyi, Nanping, Fujian | FJWY | 06/05/2009 | 117.96 | 27.66 | 6 | FJWY-1–5 | FJWY-2, 5 | FJWY-1–6 |
|  | Dehua, Quanzhou, Fujian | FJQZ | 06/26/2014 | 118.24 | 25.49 | 1 | - | - | FJQZ-1 |
|  | Yangshan, Qingyuan, Guangdong | GDQY | 08/05/2019 | 112.87 | 24.82 | 8 | GDQY-1–2 | GDQY-1–2 | GDQY-1–8 |
|  | Jiaoling, Meizhou, Guangdong | GDMZ | 09/03/2019 | 116.25 | 24.64 | 7 | GDMZ-1–2 | GDMZ-1–2 | GDMZ-1–7 |
|  | Chebaling, Shaoguan, Guangdong | GDSG | 05/01/2016 | 114.26 | 24.73 | 3 | GDSG-1 | GDSG-1 | GDSG-1–3 |
|  | Mt. Dayao, Jinxiu, Guangxi | GXJX | 09/09/2017 | 110.19 | 24.14 | 8 | GXJX-1–5 | GXJX-4 | GXJX-1–8 |
|  | Mt. Darong, Yulin, Guangxi | GXYL | 08/19/2018 | 110.28 | 22.85 | 6 | GXYL-1–5 | GXYL-2–5 | GXYL-1–6 |
|  | Jinping, Qiandongnan, Guizhou | GZJP | 08/28/2018 | 107.78 | 27.21 | 14 | GZJP-1–5 | GZJP-1–5 | GZJP-1–14 |
|  | Liping, Qiandongnan, Guizhou | GZLP | 08/12/2012 | 109.13 | 26.23 | 9 | GZLP-1–5 | GZLP-1–5 | GZLP-1–9 |
|  | Leishan, Qiandongnan, Guizhou | GZLS | 07/26/2018 | 108.28 | 26.44 | 24 | GZLS-1–5 | GZLS-1–5 | GZLS-1–24 |
|  | Maolan, Qiandongnan, Guizhou | GZML | 06/11/2018 | 107.94 | 25.32 | 1 | GZML-1 | - | GZML-1 |
|  | Mt. Yuntai, Shibing, Guizhou | GZSB | 06/28/2018 | 108.11 | 27.10 | 18 | GZSB-1–5 | GZSB-1–5 | GZSB-1–18 |
|  | Ciping, Jinggangshan, Jiangxi | JXJG | 08/15/2018 | 114.17 | 26.57 | 28 | JXJG-1–5 | JXJG-1–5 | JXJG-1–28 |
|  | Mt. Mei, Nanchang, Jiangxi | JXNC | 08/14/2018 | 115.76 | 28.79 | 16 | JXNC-1–5 | JXNC-1–5 | JXNC-1–16 |
|  | Mt. Zhougong, Ya'an, Sichuan | SCYA | 08/24/2018 | 103.02 | 29.95 | 2 | SCYA-1–2 | - | SCYA-1–2 |
|  | Taizhong, Taiwan | TWTZ | 09/09/2017 | 120.68 | 24.14 | 4 | TWTZ-1–2 | - | TWTZ-1–4 |
|  | Nantou, Taiwan | TWNT | 11/11/2010, 06/27/2012 | 120.67 | 23.92 | 5 | TWNT-1–2 | - | TWNT-1–5 |
|  | Pingdong, Taiwan | TWPD | 06/24/2012 | 120.82 | 22.15 | 2 | TWPD-1 | - | TWPD-1–2 |
|  | Zhoucun, Quzhou, Zhejiang | ZJZC | 08/12/2018 | 118.49 | 28.30 | 24 | ZJZC-1–5 | ZJZC-1–5 | ZJZC-1–24 |
|  | Bawangling, Changjiang, Hainan | HNBW | 10/21/2007 | 109.08 | 19.12 | 2 | HNBW-1–2 | HNBW-1–2 | HNBW-1–2 |
|  | Shaping, Changde, Hunan | HNCD | 04/09/2016 | 111.70 | 29.03 | 2 | HNCD-1–2 | - | HNCD-1–2 |
| *Sclomina pallens* | Langping, Tianlin, Guangxi | GXTL | 08/22/2017 | 106.36 | 24.48 | 12 | GXTL-1–12 | GXTL-1–6, 8, 10, 12 | - |
|  | Xianheping, Anlong, Guizhou | GZAL | 10/01/2017 | 105.61 | 24.99 | 5 | GZAL-1–5 | GZAL-2, 5 | - |
| *Sclomina xingrensis* | Lishuping, Xingren, Guizhou | GZXR | 10/03/2017 | 105.19 | 25.40 | 22 | GZXR-1–22 | GZXR-1, 4– 6, 8, 10–17, 21–22 | - |
| *Sclomina parva* | Hongjunlin, Yangxian, Shanxi | SXYX | 08/06/2018 | 107.52 | 33.61 | 11 | SYYX-1–11 | SYYX-1–3, 5–10 | - |
| *Sclomina guangxiensis* | Nonggang, Longzhou, Guangxi | GXLZ | 08/22/2017 | 106.96 | 22.47 | 9 | GXLZ-1–9 | GXLZ-2, 5–9 | - |
| Total |  |  |  |  |  | 254 | 131 | 95 | 195 |

**Table S2.** Primers used in this study.

| Targeted regions | Primers | Primer sequence (5’-3’) |
| --- | --- | --- |
| Mitogenome *Cytb-ND1* | CBN1F1 | CCAAGGATTAACATTTTATCCTATT |
|  | CBN1F2 | ATAATACTAACTTGAATTGGGGCCAAAC |
|  | CBN1F3 | ATTTGCCTATGCTATCTTACGATCCATTCC |
|  | CBN1R1 | ACTTTTCTAATTTTTCTTTTTATTTGAG |
|  | CBN1R2 | TTGTTGAATTTCTTCTTGTTTAGCGG |
|  | CBN1R3 | TACGTTCGGTAGCTCAAACAATTTCTTAT |
| 5.8S rRNA-ITS 2-28S rRNA | 5.8S-ITS2-28S-F | AGACTCCTTGGTCCGTGTTTC |
|  | 5.8S-ITS2-28S-R | ATCACTCGGCTCGTGGATCG |

The primers were synthesized by Sangon Biotech (Shanghai, China). The PCR reactions were performed in a 25-μL volume using EmeraldAmp Max PCR Master Mix (Takara, San Jose, USA). The PCR conditions were as follows: initial denaturation at 95 °C for 2 min, denaturation at 98 °C for 10 s, annealing at 45 °C for 50 s, and elongation at 68 °C for 1 min, followed by 40 cycles of amplification, and a final elongation cycle at 72 °C for 2 min. The PCR products were checked by agarose gel electrophoresis (1%) and sequenced on forward and reverse strands by Rui Biotech (Beijing, China).

**Table S3.** Best-fit schemes and substitution models.

| Dataset | Subset | Best model | Partition |
| --- | --- | --- | --- |
| Mitogenome | 1 | GTR+I+G | *lrRNA, ATP8_pos3, ND6_pos2, COX3_pos3, ND1_pos3, srRNA, ND3_pos1, ND2_pos1, ATP6_pos2, COX3_pos2, ND2_pos2, COX2_pos2, ND5_pos2, COI1_pos2, COX2_pos1, ATP8_pos1, ATP8_pos2, ND4L_pos2, ND4L_pos1, ND1_pos2, ND5_pos3, ND3_pos2, COX3_pos1, trRNA, ND5_pos1* |
|  | 2 | GTR+I+G | *ND2_pos3, ND1_pos1, ND4_pos1, ND4_pos3, CYTB_pos3, COI1_pos3, ND4_pos2, ATP6_pos1, ND4L_pos3, COI1_pos1, CYTB_pos2, ND3_pos3, ND6_pos3, ATP6_pos3, ND6_pos1, COX2_pos3, CYTB_pos1* |
| rRNA gene | 1 | GTR+I+G | *18S*-*ITS1*-*5.8S*-*ITS2*-*28S* |

**Table S4.** Priors of the parameters for different scenarios in DIYABC analyses.

| Type | Parameter | Step 1 | Step 2 | Step 3 |
| --- | --- | --- | --- | --- |
| Population size | N1 (*S. erinacea*) | 10–450000 | 10–450000 | 10–450000 |
|  | N2 (*S. pallens*) | 10–1200000 | 10–1200000 | 10–1200000 |
|  | N3 (*S. guangxiensis*) | 10–90000 | 10–90000 | 10–90000 |
|  | N4 (*S. parva*) | - | 10–90000 | 10–90000 |
|  | N5 (*S. xingrensis*) | - | - | 10–50000 |
|  | NA | 10–1600000 | 10–1600000 | 10–1600000 |
| Time of event | t | 10–4000000 | 10–4000000 | 10–4000000 |
|  | t1 | 10–2000000 | 10–2000000 | 10–2000000 |
|  | t2 | - | 10–1500000 | 10–1500000 |
|  | t3 | - | - | 10–1500000 |
| Admixture rate | ra | - | 0.001–0.999 | 0.001–0.999 |

**Table S5.** Pairwise uncorrected *p*-distance of five *Sclomina* species.

| Species | *S. erinacea* | *S. pallens* | *S. parva* | *S. xingrensis* | *S. guangxiensis* |
| --- | --- | --- | --- | --- | --- |
| *S. erinacea* | 0.84% |  |  |  |  |
| *S. pallens* | 5.74% | 0.93% |  |  |  |
| *S. parva* | 5.80% | 5.28% | 0.03% |  |  |
| *S. xingrensis* | 5.65% | 5.13% | 7.80% | 0.22% |  |
| *S. guangxiensis* | 8.15% | 7.88% | 9.41% | 8.57% | 0.00% |

**Table S6.** Pairwise uncorrected *p*-distance of different populations of *Sclomina erinacea* based on DNA barcoding.

| Code | AHFY | FJQZ | FJWY | GDMZ | GDQY | GDSG | GXJX | GXYL | GZJP | GZLP | GZLS | GZML | GZSB | HNBW | HNCD | JXJG | JXNC | SCYA | TWTZ | TWNT | TWPD | ZJZC |
| --- | --- | --- | --- | --- | --- | --- | --- | --- | --- | --- | --- | --- | --- | --- | --- | --- | --- | --- | --- | --- | --- | --- |
| AHFY | 0.46 |  |  |  |  |  |  |  |  |  |  |  |  |  |  |  |  |  |  |  |  |  |
| FJQZ | 0.45 |  |  |  |  |  |  |  |  |  |  |  |  |  |  |  |  |  |  |  |  |  |
| FJWY | 0.44 | 0.46 | 0.10 |  |  |  |  |  |  |  |  |  |  |  |  |  |  |  |  |  |  |  |
| GDMZ | 0.14 | 0.27 | 0.32 | 0.85 |  |  |  |  |  |  |  |  |  |  |  |  |  |  |  |  |  |  |
| GDQY | 0.18 | 0.55 | 0.63 | 0.12 | 0.64 |  |  |  |  |  |  |  |  |  |  |  |  |  |  |  |  |  |
| GDSG | 0.32 | 0.57 | 0.72 | 0.17 | 0.03 | 1.56 |  |  |  |  |  |  |  |  |  |  |  |  |  |  |  |  |
| GXJX | 0.10 | 0.60 | 0.70 | 0.26 | 0.15 | 0.36 | 0.47 |  |  |  |  |  |  |  |  |  |  |  |  |  |  |  |
| GXYL | 0.37 | 0.91 | 1.02 | 0.55 | 0.33 | 0.59 | 0.05 | 0.25 |  |  |  |  |  |  |  |  |  |  |  |  |  |  |
| GZJP | 0.05 | 0.46 | 0.57 | 0.15 | 0.18 | 0.29 | 0.13 | 0.44 | 0.06 |  |  |  |  |  |  |  |  |  |  |  |  |  |
| GZLP | 0.19 | 0.61 | 0.71 | 0.22 | 0.18 | 0.30 | 0.20 | 0.46 | 0.14 | 0.33 |  |  |  |  |  |  |  |  |  |  |  |  |
| GZLS | 0.06 | 0.38 | 0.49 | 0.13 | 0.21 | 0.32 | 0.14 | 0.45 | 0.01 | 0.16 | 0.11 |  |  |  |  |  |  |  |  |  |  |  |
| GZML | 0.05 | 0.46 | 0.56 | 0.15 | 0.20 | 0.30 | 0.13 | 0.44 | 0.00 | 0.14 | 0.01 |  |  |  |  |  |  |  |  |  |  |  |
| GZSB | 0.05 | 0.50 | 0.55 | 0.13 | 0.14 | 0.22 | 0.12 | 0.39 | 0.03 | 0.14 | 0.05 | 0.04 | 0.59 |  |  |  |  |  |  |  |  |  |
| HNBW | 2.11 | 2.21 | 2.66 | 2.05 | 1.82 | 1.87 | 2.04 | 2.31 | 2.03 | 2.00 | 2.06 | 2.05 | 1.97 | 0.00 |  |  |  |  |  |  |  |  |
| HNCD | 0.05 | 0.47 | 0.57 | 0.07 | 0.03 | 0.05 | 0.08 | 0.34 | 0.01 | 0.05 | 0.01 | 0.00 | 0.04 | 1.74 | 0.77 |  |  |  |  |  |  |  |
| JXJG | 0.19 | 0.18 | 0.34 | 0.04 | 0.19 | 0.22 | 0.28 | 0.57 | 0.19 | 0.28 | 0.15 | 0.20 | 0.21 | 2.06 | 0.09 | 0.44 |  |  |  |  |  |  |
| JXNC | 0.35 | 0.22 | 0.33 | 0.17 | 0.45 | 0.46 | 0.50 | 0.82 | 0.37 | 0.51 | 0.29 | 0.36 | 0.40 | 2.25 | 0.37 | 0.11 | 0.43 |  |  |  |  |  |
| SCYA | 1.92 | 2.55 | 2.33 | 1.86 | 1.69 | 1.65 | 1.88 | 1.98 | 2.03 | 1.97 | 2.07 | 2.05 | 1.46 | 3.40 | 1.58 | 2.10 | 2.42 | 0.00 |  |  |  |  |
| TWTZ | 3.08 | 3.31 | 3.43 | 2.93 | 2.96 | 2.74 | 3.22 | 3.47 | 3.12 | 3.15 | 3.06 | 3.14 | 2.88 | 3.57 | 2.88 | 2.98 | 3.20 | 3.40 | 0.08 |  |  |  |
| TWNT | 2.61 | 2.84 | 2.96 | 2.45 | 2.43 | 2.17 | 2.72 | 2.95 | 2.65 | 2.64 | 2.58 | 2.67 | 2.40 | 3.02 | 2.37 | 2.50 | 2.74 | 2.85 | 0.28 | 0.68 |  |  |
| TWPD | 2.84 | 3.07 | 3.19 | 2.67 | 2.66 | 2.34 | 2.95 | 3.18 | 2.88 | 2.84 | 2.82 | 2.90 | 2.63 | 3.42 | 2.60 | 2.74 | 2.97 | 3.08 | 0.78 | 0.25 | 0.46 |  |
| ZJZC | 0.44 | 0.44 | 0.00 | 0.30 | 0.62 | 0.70 | 0.68 | 0.99 | 0.56 | 0.70 | 0.48 | 0.56 | 0.54 | 2.62 | 0.57 | 0.32 | 0.31 | 2.31 | 3.43 | 2.96 | 3.19 | 0.18 |

**Table S7.** The scenario selection and model checking results of each step in the DIYABC analyses.

| Step | Number of scenarios | Selected scenario | Probability of selected scenario | Total number of summary statistics | Number of summary statistics with P < 0.05 or P > 0.95 |
| --- | --- | --- | --- | --- | --- |
| 1 | 9 | 3 | 0.3497 [0.3317, 0.3676] | 39 | 0 |
| 2 | 8 | 3 | 0.6266 [0.5662, 0.6870] | 62 | 0 |
| 3 | 14 | 4 | 0.3147 [0.2342,0.3953] | 90 | 0 |

**Table S8.** The environmental variable contribution to the Maxent model in ENM analyses for present, LGM and LIG periods.

| Variable | Present | LGM | LIG |
| --- | --- | --- | --- |
| BIO1 | 6.1 | 8.9 | 3.8 |
| BIO2 | 12.1 | 11.8 | 10.9 |
| BIO3 | 21.3 | 20.5 | 22.0 |
| BIO5 | 0.0 | 0.1 | 0.2 |
| BIO8 | 1.5 | 1.6 | 1.8 |
| BIO13 | 40.2 | 37.9 | 41.6 |
| BIO14 | 9.8 | 10.7 | 11.4 |
| BIO15 | 9 | 8.4 | 8.2 |

**Table S9.** Pairwise genetic differentiation *F_st_* values of different populations of *Sclomina erinacea*.

| Code | AHFY | FJWY | GDMZ | GDQY | GDSG | GXJX | GXYL | GZJP | GZLP | GZLS | GZSB | HNBW | HNCD | JXJG | JXNC | SCYA | TW | TWNT | TWPD | ZJZC |
| --- | --- | --- | --- | --- | --- | --- | --- | --- | --- | --- | --- | --- | --- | --- | --- | --- | --- | --- | --- | --- |
| AHFY | 0.0000 |  |  |  |  |  |  |  |  |  |  |  |  |  |  |  |  |  |  |  |
| FJWY | 0.5907 | 0.0000 |  |  |  |  |  |  |  |  |  |  |  |  |  |  |  |  |  |  |
| GDMZ | 0.3165 | 0.2615 | 0.0000 |  |  |  |  |  |  |  |  |  |  |  |  |  |  |  |  |  |
| GDQY | 0.3229 | 0.5304 | 0.2792 | 0.0000 |  |  |  |  |  |  |  |  |  |  |  |  |  |  |  |  |
| GDSG | 0.3825 | 0.6191 | 0.3512 | 0.1326 | 0.0000 |  |  |  |  |  |  |  |  |  |  |  |  |  |  |  |
| GXJX | 0.1062 | 0.4427 | 0.2395 | 0.1596 | 0.2402 | 0.0000 |  |  |  |  |  |  |  |  |  |  |  |  |  |  |
| GXYL | 0.5052 | 0.7482 | 0.4641 | 0.3155 | 0.4703 | 0.1028 | 0.0000 |  |  |  |  |  |  |  |  |  |  |  |  |  |
| GZJP | 0.3011 | 0.7569 | 0.5730 | 0.5240 | 0.6253 | 0.2967 | 0.6593 | 0.0000 |  |  |  |  |  |  |  |  |  |  |  |  |
| GZLP | 0.2471 | 0.7154 | 0.4930 | 0.4399 | 0.5266 | 0.2331 | 0.5993 | 0.2660 | 0.0000 |  |  |  |  |  |  |  |  |  |  |  |
| GZLS | 0.4636 | 0.8120 | 0.6738 | 0.6353 | 0.7307 | 0.4253 | 0.7432 | 0.1404 | 0.4218 | 0.0000 |  |  |  |  |  |  |  |  |  |  |
| GZSB | 0.1685 | 0.5778 | 0.4211 | 0.3857 | 0.4618 | 0.1735 | 0.4789 | 0.1200 | 0.1701 | 0.2941 | 0.0000 |  |  |  |  |  |  |  |  |  |
| HNBW | 0.8607 | 0.9540 | 0.8356 | 0.8136 | 0.7592 | 0.7466 | 0.9081 | 0.9168 | 0.8935 | 0.9406 | 0.8285 | 0.0000 |  |  |  |  |  |  |  |  |
| HNCD | 0.1044 | 0.6747 | 0.2367 | 0.0278 | -0.0015 | -0.0223 | 0.4431 | 0.3388 | 0.2578 | 0.5554 | 0.0714 | 0.8397 | 0.0000 |  |  |  |  |  |  |  |
| JXJG | 0.3728 | 0.2023 | 0.0904 | 0.3370 | 0.4453 | 0.3456 | 0.4781 | 0.5520 | 0.5081 | 0.6194 | 0.4679 | 0.8443 | 0.3149 | 0.0000 |  |  |  |  |  |  |
| JXNC | 0.6185 | 0.3947 | 0.2885 | 0.5802 | 0.6843 | 0.5176 | 0.7308 | 0.7446 | 0.7166 | 0.7906 | 0.6102 | 0.9334 | 0.6692 | 0.1354 | 0.0000 |  |  |  |  |  |
| SCYA | 0.7514 | 0.9261 | 0.7179 | 0.6725 | 0.6061 | 0.5189 | 0.8421 | 0.8499 | 0.8115 | 0.8924 | 0.6402 | 0.9647 | 0.7214 | 0.7339 | 0.8885 | 0.0000 |  |  |  |  |
| TW | 0.8999 | 0.9614 | 0.8735 | 0.8558 | 0.8498 | 0.8033 | 0.9306 | 0.9305 | 0.9158 | 0.9478 | 0.8572 | 0.9700 | 0.9127 | 0.8637 | 0.9422 | 0.9722 | 0.0000 |  |  |  |
| TWNT | 0.7733 | 0.8392 | 0.7714 | 0.7649 | 0.7041 | 0.7231 | 0.8157 | 0.8637 | 0.8256 | 0.9022 | 0.8011 | 0.7862 | 0.7133 | 0.8216 | 0.8805 | 0.7690 | 0.3231 | 0.0000 |  |  |
| TWPD | 0.8552 | 0.9449 | 0.8341 | 0.8130 | 0.7484 | 0.7538 | 0.9033 | 0.9164 | 0.8926 | 0.9406 | 0.8345 | 0.9326 | 0.8158 | 0.8493 | 0.9331 | 0.9343 | 0.8570 | 0.2466 | 0.0000 |  |
| ZJZC | 0.5204 | -0.0161 | 0.2362 | 0.5317 | 0.6290 | 0.4952 | 0.6490 | 0.6804 | 0.6463 | 0.7291 | 0.5825 | 0.8943 | 0.5628 | 0.2023 | 0.2804 | 0.8205 | 0.9064 | 0.8628 | 0.8966 | 0.0000 |

**Table S10.** Results of Multiple Matrix Regression with Randomization (MMRR) analysis and Mantel test for *Sclomina erinacea*.

| Type | MMRR |  |  | Mantel test |  |
| --- | --- | --- | --- | --- | --- |
|  | *R^2^* (*p*) | *β_D_* (*p*) | *β_E_* (*p*) | *r_D_* (*p*) | *r_E_* (*p*) |
| Model | 0.471 | - | - | - | - |
| IBD | - | 0.406 (0.001) | - | 0.405 (0.001) | - |
| IBE | - | - | 0.388 (0.001) | - | 0.461 (0.002) |

IBD, Isolation by distance; IBE, Isolation by environment.

**Table S11.** GenBank accessions of mitogenomes and rRNA genes obtained in this study.

| **Species** | **Mitogenomes** | **GenBank accessions** | **Nuclear rRNA genes** | **GenBank accessions** |
| --- | --- | --- | --- | --- |
| *S. erinacea* | AHFY-1–5 | ON116694–ON116698 | AHFY-1–5 | ON130390–ON130394 |
|  | FJQZ-1 | ON116699 | - | - |
|  | FJWY-1–6 | ON116700–ON116705 | FJWY-2, 5 | ON130395–ON130396 |
|  | GDMZ-1–7 | ON116706–ON116712 | GDMZ-1–2 | ON130397–ON130398 |
|  | GDQY-1–8 | ON116713–ON116720 | GDQY-1–2 | ON130399–ON130400 |
|  | GDSG-1–3 | ON116721–ON116723 | GDSG-1 | ON130401 |
|  | GXJX-1–8 | ON116724–ON116731 | GXJX-4 | ON130402 |
|  | GXYL-1–6 | ON116732–ON116737 | GXYL-2–5 | ON130420–ON130423 |
|  | GZJP-1–14 | ON116738–ON116751 | GZJP-1–5 | ON130432/ON130438, ON130424–ON130426 |
|  | GZLP-1–9 | ON116752–ON116760 | GZLP-1–5 | ON130427–ON130431 |
|  | GZLS-1–24 | ON116761–ON116784 | GZLS-1–5 | ON130433–ON130437 |
|  | GZML-1 | ON116785 | - | - |
|  | GZSB-1–18 | ON116786–ON116803 | GZSB-1–5 | ON130439–ON130443 |
|  | HNBW-1–2 | ON116804–ON116805 | HNBW-1–2 | ON130459–ON130460 |
|  | HNCD-1–2 | ON116806–ON116807 | - | - |
|  | JXJG-1–28 | ON116808–ON116835 | JXJG-1–5 | ON130461–ON130465 |
|  | JXNC-1–16 | ON116836–ON116851 | JXNC-1–5 | ON130467–ON130471 |
|  | SCYA-1–2 | ON116852–ON116853 | - | - |
|  | TWNT-1–5 | ON116854–ON116858 | - | - |
|  | TWPD-1–2 | ON116859–ON116860 | - | - |
|  | TWTZ-1–4 | ON116861–ON116864 | - | - |
|  | ZJZC-1–24 | ON116865–ON116888 | ZJZC-1–5 | ON130466, ON130481–ON130484 |
| *S. guangxiensis* | GXLZ-1–9 | ON116889–ON116897 | GXLZ-2, 5–9 | ON130403–ON130408 |
| *S. pallens* | GZAL-1–5 | ON116898–ON116902 | GZAL-2, 5 | ON130418–ON130419 |
|  | GXTL-1–12 | ON116903–ON116914 | GXTL-1–6, 8, 10, 12 | ON130409–ON130417 |
| *S. parva* | SYYX-1–11 | ON116915–ON116925 | SYYX-1–3, 5–10 | ON130472–ON130480 |
| *S. xingrensis* | GZXR-1–22 | ON116926–ON116947 | GZXR-1, 4– 6, 8, 10–17, 21–22 | ON130444–ON130458 |
